# Acceptance of Industrial Collaborative Robots by People With Disabilities in Sheltered Workshops

**DOI:** 10.3389/frobt.2020.541741

**Published:** 2021-01-11

**Authors:** Sandra Drolshagen, Max Pfingsthorn, Pascal Gliesche, Andreas Hein

**Affiliations:** ^1^R&D Division Manufacturing, OFFIS Institute for Information Technology, Oldenburg, Germany; ^2^R&D Division Health, OFFIS Institute for Information Technology, Oldenburg, Germany; ^3^Assistance Systems and Medical Device Technology, Department of Health Services Research, Carl von Ossietzky Universität Oldenburg, Oldenburg, Germany

**Keywords:** robotics, disabled, human-robot collaboration, inclusion, sheltered workshop, acceptance

## Abstract

The integration of people with disabilities into the working world is an important, yet challenging field of research. While different inclusion efforts exist, people with disabilities are still under-represented in the open labor market. This paper investigates the approach of using a collaborative robot arm to support people with disabilities with their reintegration into the workplace. However, there is currently little literature about the acceptance of an industrial robot by people with disabilities and in cases where a robot leads to stress, fear, or any other form of discomfort, this approach is not feasible. For this reason, a first user study was performed in a sheltered workshop to investigate the acceptance of a robot arm by workers with disabilities. As a first step in this underdeveloped field, two main aspects were covered. Firstly, the reaction and familiarization to the robot arm within a study situation was closely examined in order to separate any effects that were not caused by the moving robot. Secondly, the reaction toward the robot arm during collaboration was investigated. In doing so, five different distances between the robot arm and the participants were considered to make collaboration in the workplace as pleasant as possible. The results revealed that it took the participants about 20 min to get used to the situation, while the robot was immediately accepted very well and did not cause fear or discomfort at any time. Surprisingly, in some cases, short distances were accepted even better than the larger distances. For these reasons, the presented approach showed to promise for future investigations.

## Introduction

The World Health Organization (WHO) estimates that ~15% of the world's population lives with some form of disability. This equates to more than a billion people, with an estimation, that more than 650 million of these are of working age. However, analysis conducted by the WHO of 51 countries showed that only 52.8% of men and 19.6% of women with disabilities have employment. This is a significant difference compared to the employment rates of non-disabled people. The underrepresentation of employees with disabilities in the working world, exists for many reasons, including productivity differentials, discrimination, and prejudice on part of both employers and employees. However, finding employment is an important aspect for integration of people with disabilities into society and thereby increasing their quality of life (World Health Organization, 2011).

It is not only people with disabilities who would benefit from being integrated into work more easily. Sheltered workshops (SWs) and companies would also profit. SWs, in Germany for example, are non-profit organizations which help people with disabilities to find work and enter or re-enter the labor market. However, even though they are non-profit, SWs must compete in the open market. For this reason, they need to be efficient to stay competitive (Miralles et al., 2008). This can lead them to try to retain their most productive workers instead of losing them to the open labor market— <1% of sheltered workshop employees enter the labor market afterwards (Hoock, 2017). Moreover, due to staff shortage, SWs cannot always meet each worker's needs as required to improve their abilities. Another crucial aspect is the change of the industrial environment caused by the so-called fourth industrial revolution “Industry 4.0.” With the help of digitalization, the trend in manufacturing will shift toward individual products and thus small batch sizes (Mark et al., 2019). Since this process will likely also affect SWs, demanding greater flexibility and agility from their employees will likely become unavoidable. Apart from SWs, companies could also use support to be able to incorporate disabled workers efficiently in their processes. The productivity aspect is even more important for them, but at the same time the German government, for example, requires companies to employ at least 5% of people with disabilities. High fees are due in case of violation (Neuhaus, 2014). For both businesses and employees, it is important to find a solution that helps train a wider range of people without increasing the need for considerably more supervisors. Eventually, this will benefit workers with disabilities, as it will help more people with disabilities to find work and make it easier for them to enter the open labor market. This will improve their quality of life, make them feel integrated into society, and provide financial security (World Health Organization, 2011). Also, the United Nations Convention on the Rights of Persons with Disabilities presents many aspects highlighting the importance of this field (UN General Assembly, 2006). Finally, the economy profits from these changes as well, because the increased number of employees means higher productivity and more taxes can be collected (World Health Organization, 2011).

**Figure 1:**
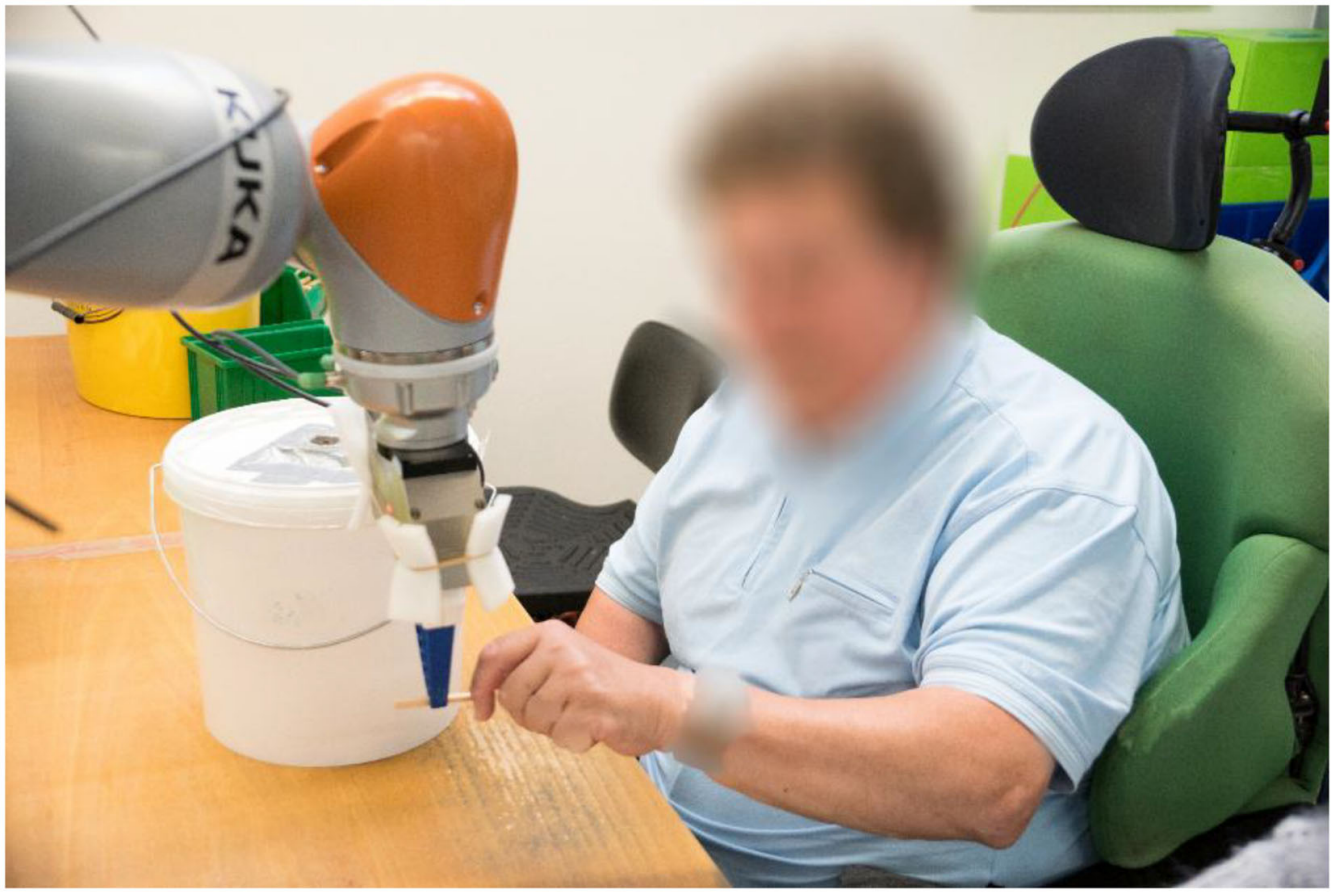
Collaboration between a robot arm and a human with a disability.

To achieve these goals, people with disabilities must be supported to be more flexible in terms of their work tasks and familiarize themselves more quickly with new production steps. One possibility for achieving this is the use of assistive collaborative robots (consult Figure 1 for an example). The robot would be able to render assistance to the people with disabilities as soon as they need help or specific training. In addition, the robot could help to perform certain tasks together with the worker in order to teach a specific movement which would bring a rehabilitative benefit. This would also ensure a safe execution of the task (e.g., with heavy loads).

The use of collaborative robots (cobots) in industry is already widely explored, so it seems to be a logical consequence to also use them to support people with disabilities. However, while the interaction of humans and robots is well-researched, little is known about how adults with disabilities would react in collaboration with an industrial cobot. People with disabilities are thought to be more sensitive to fast and loud movements or changing conditions, and all of these can be caused by a robot (Bosch Media Service, 2018). In case a robot leads to stress, fear, or any other form of discomfort, their use in SWs is not feasible. Therefore, this paper presents a first user study in a SW, with the aim of investigating the acceptance of a robot arm by workers with disabilities.

Here it should be noted that the term “disability” describes a broad range. People with disabilities are diverse and varied. Their health conditions can be visible or invisible, temporary or long term, some people are in pain while others consider themselves to be in excellent health. People may have a physical disability, a mental disability or both at the same time, and the severity can vary enormously (World Health Organization, 2011). To include all these aspects can be challenging. Therefore, Holloway (2019) proposes the DIX (disability interaction), an open-source community, where assistive systems are developed. Assistive technologies for people with disabilities can become universal and, ultimately, benefit inclusion, encourage innovation, and add value for societies. This is demonstrated by examples from history, such as the typewriter or commercial email client, which were both developed to help deaf and blind people to communicate (Holloway, 2019).

Following this idea, the concept presented here might later help people with varying degrees of disability, as well as healthy people. Hence, it might help both the SWs and open labor market. However, as a first step, the focus of this paper was put on SWs and thus severely disabled people. People with less severe disabilities are often already integrated in industry, so the focus is on the large population currently unable to move into the labor market. Another aspect that was considered is that people with disabilities often demand a high degree of consistency and are slow to adapt to new situations (Clark, 2000). For this reason, the time they need to get used to the study situation was evaluated as well.

The main contributions of this paper are thus:

- Examining the familiarization process of a person with a disability with a monitoring situation and a robot arm
- Analyzing the overall acceptance and limitations of a collaboration between an industrial robot arm and a person with a disability
- Investigating the effect of the spatial distance between a robot arm and a human participant

The corresponding findings can be summarized as follows:

- It takes the participants about 20 min to get used to the study situation and the robot
- The robot was immediately well-accepted and did not cause fear or discomfort at any time
- Short distances were accepted as well, and in some cases even better than larger distances.

In the following sections, the state of the art is described first, including the deficiencies. Secondly, the method is described, followed by an evaluation of both parts of the study—the familiarization phase and the human robot collaboration. Finally, all results are discussed.

## Related Work

In this paper, two research areas are combined. The use of cobots in manufacturing and the desire to support workers with disabilities to be able to conduct a job. In the following, relevant literature of both fields, as well as first publications on overlapping topics, are presented.

### Workers With Disabilities

Mark et al. (2019) states the importance of integrating people with disabilities into the labor market, especially now the fourth industrial revolution is taking place. In this context, different assistant technologies, such as sensorial aid systems, physical aid systems, and cognitive aid systems are discussed, albeit not empirically examined (Mark et al., 2019). Besides these rather theoretical considerations, there exists already some realized research on the integration of people with disabilities into the labor market. Eriksson and Ortega (2006) suggest, for example, the use of job rotation to increase the employees' abilities by exposing them to different tasks. This will also improve the knowledge of the employer, helping them to recognize the talents of each employee (Eriksson and Ortega, 2006). Works, such as Miralles et al. (2008) and Costa and Miralles (2009), go one step further. They deal with the question of how to schedule the rotation in a SW in such a way that workers can improve their abilities while still respecting desired productivity levels of the SW. This is important for the SW in order to stay competitive since every worker might have different skills and thus have different execution times of the same task, while others may be completely incapable of executing a certain task (Costa and Miralles, 2009).

Apart from these strategies and other reasonable accommodations such as making existing facilities accessible, providing qualified readers or interpreters, etc. (EEOC, 2002), using technology to help people with disabilities is not a completely new approach. Assistive systems for industrial workplaces are already being investigated in many situations, especially their use for elderly and people with disabilities. A rather simple example is the “pick-by-light” approach (de Vries et al., 2016). During the assembly of a certain task, different tools and assembly parts can be found in different boxes. The box to pick from is highlighted by a light and the pick is then controlled by light barriers. Moreover, the assembly process is often visualized on a monitor. Apart from the picking control, no feedback is given to the user, as summarized by de Vries et al. (2016). Korn et al. (2013) go one step further. In order to provide the workers with additional cognitive support, they project the work-relevant information directly onto the workspace (*in-situ* projection). Whenever a working step is completed, the worker presses a green button to get to the next step. The *in-situ* projection, however, has been shown to worsen performance of severely impaired workers (Korn et al., 2013). Korn et al. (2015) presents a Context-Aware Assistive System (CAAS) to give real-time feedback such as knowing which step comes next or detecting errors. This is done by using motion data. The CAAS is a system consisting of a Kinect depth-camera to capture touch with the workplace surface, a Leap Motion that detects hand movements, and a web-camera to identify currently used tools and assembly parts. Detected motion trajectories are then compared to reference-trajectories. This information is used to eventually adjust the speed of production or the number of steps to be assembled by the person. Moreover, the scope of the instructions can be adapted, or visual or audio feedback can be given. It could also be determined whether the worker reduces work speed because of boredom or exhaustion by reflecting the emotional state (e.g., nervous hands, facial expression, etc.), although this is not implemented in Korn et al. (2015).

### Robots for People With Disabilities

Apart from the aforementioned assistive systems, more recent publications investigate the use of robots to help people with disabilities at work. One example is a system presented in Gräser et al. (2013) that assists paraplegic librarians at their workplace by taking over all book manipulation tasks. Another example of robots helping people with disabilities is the AQUIAS project (Kremer et al., 2018). The project aims to help people with disabilities participate in modern manufacturing by having the robots take over predefined tasks that are physically demanding. The people with disabilities perform all other steps, such as quality control. Kremer et al. (2018) investigated different experimental setups to yield a comfortable yet, at the same time, efficient workplace design. Among others, they concluded that a spatial attachment of the robot too close to the person would lead to problems of acceptance, since people with disabilities are believed to be more sensitive to fast and sudden movements and noises. However, these assumptions, in the context of robotic assistance are not based on empirical studies, but personal estimates, as verified in personal contact with the authors (Kremer et al., 2018). Kremer (2019) describes a future scenario in which robots will assist humans with disabilities at work based on the AQUIAS project.

A different aspect worth mentioning is the rehabilitative use of robots. Although the context of use is a different one, the ideas could be transferred to people with disabilities in industry. Robots in a slightly modified form, namely that of exoskeletons, are already often used for the rehabilitation of upper and lower limbs. One example is the ARMin II exoskeleton of the ETH Zürich (Zürich, Switzerland) (Nef et al., 2007). The ARMin II is used for rehabilitation of the upper limbs. Among other activities, the user can play various virtual games. One example is a game in which the user guides their hand through a virtual labyrinth. As soon as the user's hand is no longer centered on the labyrinth path but intersects with a wall, the exoskeleton exerts a force to correct the position. This supports the patient in learning to move correctly (Colledanchise and Ögren, 2017). This approach could be shifted toward robots helping a human worker, to bring a rehabilitative benefit by correcting their movements. Moreover, robot-based autism therapy, especially for children, is a rapidly growing research area. The interaction is intended to increase their social skills, along with an associated rehabilitation of motor skills (Giullian et al., 2010; Ricks and Colton, 2010; Jouaiti and Hénaff, 2019). An initial study on teaching students with intellectual disabilities different tasks using a social robot also showed promising results, probably because the students felt less pressure when asking a robot to repeat the explanation than they did with a judgmental human equivalent (Reardon et al., 2019).

### Cobots in Industry

Apart from helping workers with disabilities, robots are also frequently used to cooperate with healthy workers in industry. This section gives a brief overview of a small relevant set of current research areas in this broad field, since some aspects are applicable for humans with disabilities and thus show the potential of this field of research.

One important aspect of human-robot collaboration is to avoid unintentional collisions, but at the same time to enable intentional contacts, as Mišeikis et al. (2016) investigated. This is necessary, for example, when transferring objects. It must be ensured that the handover is as natural and pleasant for the person as possible. There is a lot of research on this subject, for example Walters et al. (2007), Sidobre et al. (2012), or Vogt et al. (2016). The latter concluded, for example, that a handover is most pleasant when approached from the side. At the same time, Bortot et al. (2013) pointed out that a robot is most readily accepted when its end effector proceeds on a straight line.

### Acceptance of Robots

To evaluate the overall acceptance of robotic assistants, different models, and evaluation approaches exist. Commonly adapted models in the field of information systems include the Technology Acceptance Model (TAM) from Davis (1989), the Unified Theory of Acceptance and Use of Technology Model (UTAUT) by Venkatesh et al. (2003), and the Chain Model of Goodhue and Thompson (1995). The former is the most widely used model of technology acceptance and proposes two main variables that indicate acceptance: perceived usefulness and perceived ease of use. While the other two models consider additional variables, none of these three is thought to be adequate to investigate robot acceptance (Beer et al., 2011). This specific research area often focuses on the use of questionnaires similar to the Negative Attitude Toward Robots Scale (NARS) by Nomura et al. (2006). The NARS assesses negative attitudes toward a robot in regard to the interaction with robots, the social influence of robots, and emotional interactions with robots (Nomura et al., 2006). Beer et al. (2011) proposes that robot acceptance is influenced by the robot's function, social ability, and form. Especially the latter two, which are not included in traditional technology acceptance models, but might play an important role for robots that are intended to socially interact with humans (Beer et al., 2011). Broadbent et al. (2009) conducted a literature review on human responses to healthcare robots and found that individual variables like the age, needs, gender, etc., as well as robot variables like appearance, size, ergonomics, etc. influence the acceptance of health robots. Furthermore, Weiss et al. (2008) argues that observation and behavioral analysis of social interaction in the real-world environment are necessary when dealing with the acceptance of robots. They also propose the use of a breaching experiment, which disrupts ordinary activities in order to detect some unusual behaviors and reactions by the users (Weiss et al., 2008). Choi et al. (2013) include considerations of intergroup relations and body zones into their research on the acceptance of vacuum cleaning robots.

## Method

The aforementioned, technological inclusion approaches each cover a single aspect of integrating people with disabilities or providing some kind of support using technology. However, some of the examples given have not yet been tested on people with disabilities, while others offer either a form of feedback or support, but not both at the same time. The combination of multiple aspects is an interesting field of research and we performed a first step. To the best of our knowledge, an industrial robot arm has not yet been tested for collaboration with an adult human with a mental and/or physical disability in a working environment. However, the reaction to this collaboration, as well as any fear or feelings of discomfort are the basis for further investigations of the use of such a system and thus subject to the presented study.

To evaluate the acceptance of an industrial robot, a study following a human-centered design was chosen. In the human-centered design, in contrast with the robot-centered or robot-cognition-centered design, the human perspective is central (Dautenhahn, 2007). This involves acceptability, believability, and overall perception of the robot by the human user (Alenljung et al., 2017). According to Weiss et al. (2011), important factors for investigating human-robot interaction are usability, social acceptance, user experience (UX), and societal impact, as explained in their USUS Evaluation Framework. However, the consideration of all of these aspects would exceed the scope of this work. In this study, the focus was placed on the UX in particular the feeling of security (Weiss et al., 2011). The aim is to analyse how a workplace collaboration between a robot arm and people with disabilities may be made most comfortable for the user. Also, the effect of distance between the robot arm and the worker's acceptance is considered, to support the worker as effectively as possible without them perceiving the robot arm as threatening at a normal working speed.

To perform an UX evaluation, a goal must be defined (Lindblom et al., 2020). For this paper, the goal was to recognize any indications of fear or discomfort regarding the robot arm. Therefore, a natural field study was conducted to observe the real UX, as proposed by Lindblom et al. (2020). To achieve this goal, the task the participants conducted was chosen such that no additional irritation was introduced, which is why the participants were already familiar with the tasks. Moreover, literature suggests that people with disabilities are more averse to change and often need a steady daily routine (Kräling, 2000; Wetzler, 2000). Merely participating in a study can already cause strong reactions that are not necessarily triggered by the actual tasks of the study, as was revealed during preceding discussions with the heads of the SW in which the study took part. For this reason, our study was split into two parts. First, a familiarization with the robot arm should be achieved and information on the training period were collected. Afterward, the interaction with the robot was considered. Using this stepwise approach of introducing the robot arm, any effects that are not caused by the moving robot arm could be eliminated.

The UX was then assessed using subjective measurements, which are especially suited for perceived trust and safety (Lindblom et al., 2020). For this evaluation, a combination of direct observation and recorded observation was used to capture all possible aspects, since commonly used questionnaires are not applicable for the special target group. This is an approach proposed by Martín Rico et al. (2020), who use observers' assessments and observations to rate the acceptance of a humanoid robot by people with dementia. They argue that commonly used questionnaires like the NARS do not always lead to reliable results that agree with observations (Martín Rico et al., 2020). In addition to these points, a qualitative measurement is preferable for this study since it is infeasible to have a huge number of participants in the SW and even then, the strong heterogeneity would make drawing statistical conclusions difficult. Furthermore, qualitative measures are considered to be better suited to early testing stages, because they allow researchers to draw feedback on the cause of the triggered action and how to fix it as proposed by Lindblom et al. (2020).

In the following sections a detailed description of the provided methods is presented.

### Apparatus and Setup

**Figure 2:**
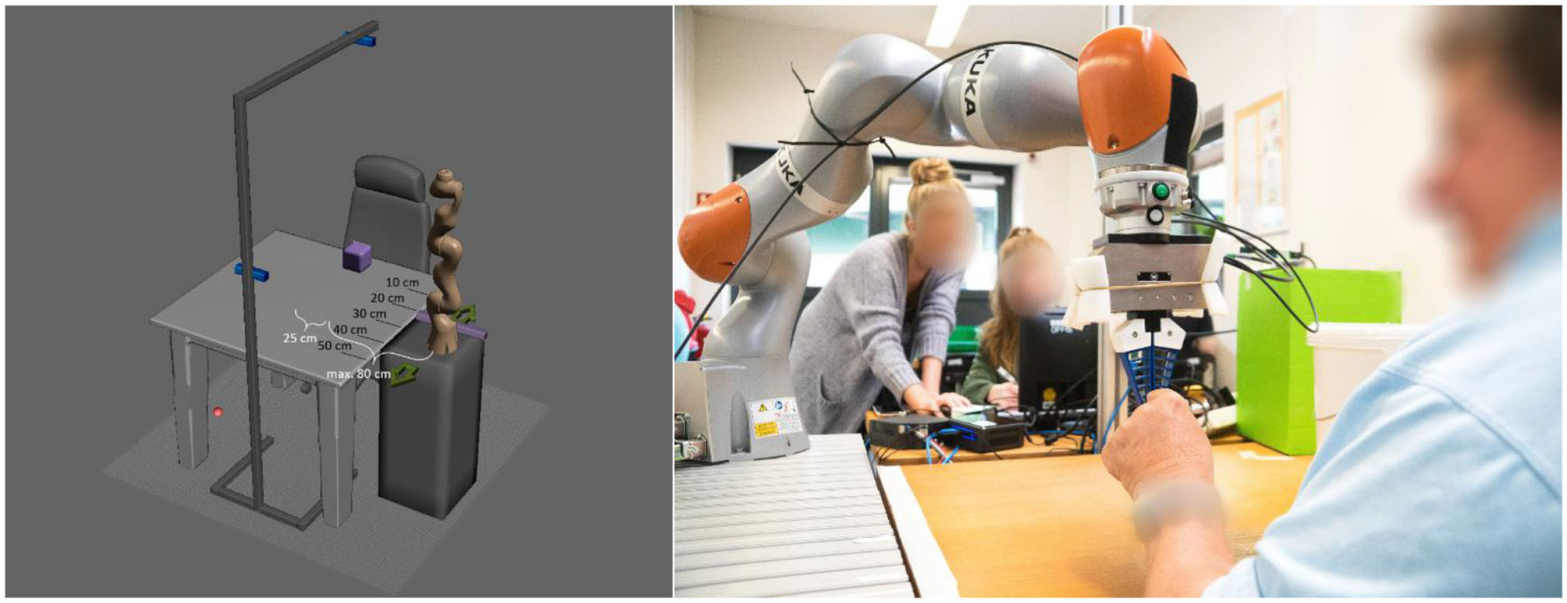
Schematic **(Left)** and actual **(Right)** setup of the study.

The study was conducted with a KUKA LBR iiwa 7 R800 of the KUKA AG (Augsburg, Germany) and a WSG50-110 Gripper from the Weiss Robotics GmbH & Co., KG (Ludwigsburg, Germany) having DHAS-GF-80-U-BU adaptive fingers of the Festo AG & Co., KG (Esslingen am Neckar, Germany) attached. All components were programmed using the Robot Operating System (ROS). The robot arm was standing on a table of 79 cm height, which equates to the height of the working desk of the workers. It was placed right next to the working desk with a displacement of about 1.05 m to the left of the participant. The robot arm could be varied to have an anterior distance between 10 and 50 cm measured from the front edge of the working desk, hereafter referred to as distances 1–5, respectively (see Figure 2). The robot's end-effector moved with a velocity of ~26 $\frac{cm}{s}$ on a linear trajectory. On the opposite side of the desk, a construction was installed containing two Realsense r200 depth-cameras of the Intel Corporation (Santa Clara, California, USA). One was at the same height as the participant's head, monitoring him or her from the front, and one was top down, monitoring the participant from above. A third Realsense r200 camera was installed on the desk for the third day, when the robot arm was moving, because the robot covers the participant in the frontal camera. All cameras monitored rgb and depth images at 15 fps. Their communication was also realized via ROS.

### Participants

The study was carried out in a SW in Oldenburg, Germany. All participants are employed in this work center and were thus familiar with the surroundings and people. The recruitment of the participants was performed by the heads of the SW, since they had the most knowledge about their employees. Workers suitable for participation were considered to be those, already familiar with the task at hand: checking the size of small wooden sticks by placing them inside a box. This requirement was made in order to have some level of homogenization among the participants. Another requirement was for the participant to have a severe disability. All participants were asked for their consent, while some additionally required the permission of their legal guardian. Altogether, 10 participants were included, seven males and three females with ages ranging from 21 to 60 years (mean = 42.3, standard deviation = 13.04). While no detailed information about the individual degree of disability could be provided, all participants had a disability between 50 and 100%. However, 80% of the employees with disabilities in the SW had a disability of 100%, so a similar distribution can be assumed for the participants of this study. Four participants had a mental disability, one a physical disability, three had both a mental disability, and a physical disability, one participant had a visual and mental impairment, and one had a mental disability and learning disorder (see Table 1 for a detailed list). No access to the exact medical diagnoses could be provided. All participants were already familiar with the required task and none of them had ever worked with a robot arm before.

**Table 1:** Information on the participants and their disabilities.

**Participant**	**Disability**
1	Physical and mental disability
2	Acquired short-term memory loss
3	Physical and mental disability
4	Physical and learning disorder
5	Physical disability
6	Mental disability
7	Acquired physical and mental disability
8	Visually and mental impairment
9	Mental disability
10	Mental disability

### Safety and Ethical Aspects

The used industrial robot arm KUKA LBR iiwa 7 R800 is designed for close collaboration with humans and is equipped with appropriate safety standards. It fulfills the required EN ISO norms e.g., EN ISO 10218-1:201 for industrial robots (KUKA, 2020). Moreover, a detailed risk analysis was performed prior to the study, which can be seen in Supplementary Table 1. The robot arm measures its forces in terms of torques at each joint. For these reasons, the robot arm was placed in such a way that it had a maximal leverage when closest to the participant, to register high torque values more easily at a short distance. To be more precise, it had a displacement of 0.8 m at maximum leverage, while still having a distance to the left of the participant of 0.25 m and anterior distances between 0.1 and 0.5 m measured from the front edge of the working desk, when closest to the participants. With the maximum torque set to 25 Nm a collision force of 31.25 N would result. However, assuming an approximate collision area of 4 cm^2^, the pressure during a collision would be 7.81 $\frac{N}{cm^{2}}$. According to the DIN-ISO/TS 15066, which specifies the safety requirements for human-robot interactions, a maximal force of 65 N and maximal pressure of 110 $\frac{N}{cm^{2}}$would be allowed even in the most sensitive area, the human face. Since our values are thus far below the upper threshold, a spatially close installation and cooperation can be considered safe even if a collision occurs with any part of the body. Furthermore, while neither the robot arm nor its fingers contain any sharp edges, the edges of the gripper were covered with foam in order to prevent any kind of injuries at contact.

This study was carried out in accordance with the recommendations of the regulations governing the principles for safeguarding good academic practice at the Carl von Ossietzky University Oldenburg, Germany, of the Commission for Research Impact Assessment and Ethics. The protocol was approved by the Commission for Research Impact Assessment and Ethics (Drs.EK/2019/038). All participants, or, if required, their legal guardian gave written informed consent in accordance with the Declaration of Helsinki.

### Task Description

The experiment was conducted on 3 subsequent days.

- Day 1: The participant carried out the task alone without the robot being present.
- Day 2: The participant carried out the task alone with the robot being present; however, the robot was turned off.
- Day 3: The participant carried out the task in collaboration with the robot.

**Figure 3:**
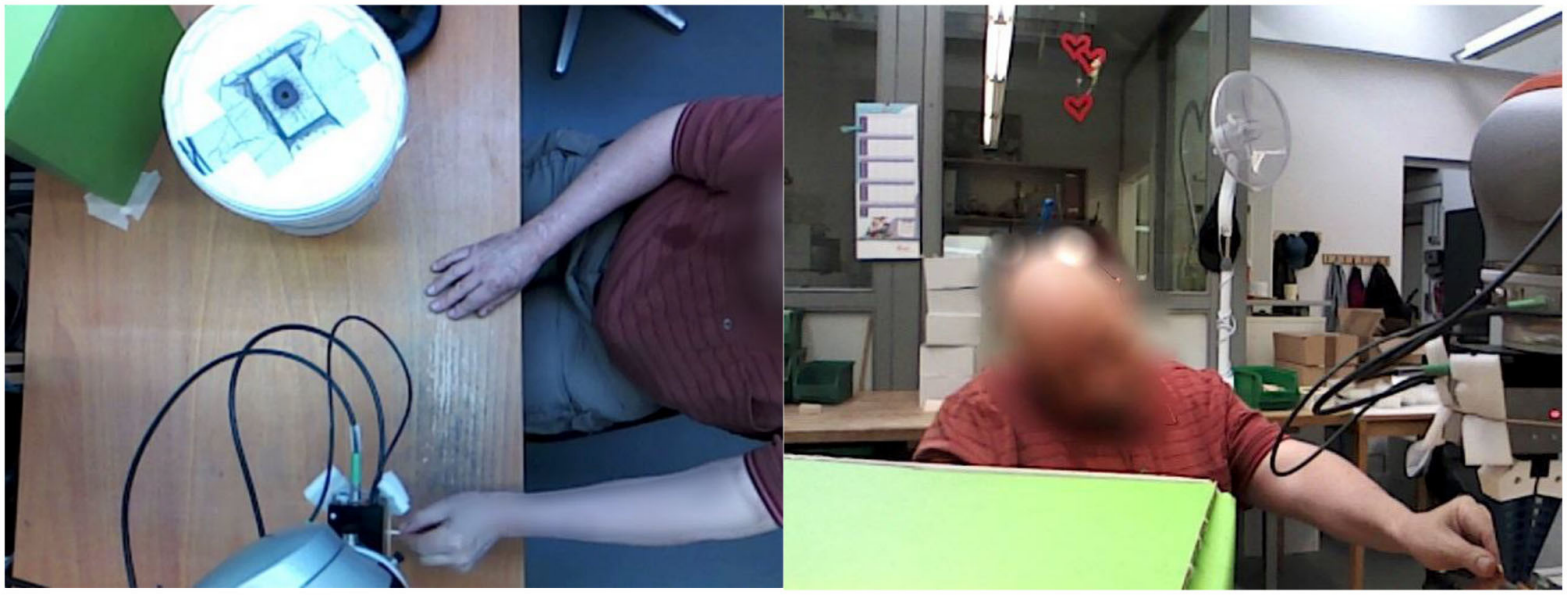
Snapshot of the participant removing the wooden stick from the robot before placing it into the white box. Top view **(Left)** and front view **(Right)**.

On each day, the participants conducted the same task, while the physical appearance and movement of the robot increased from day to day. The task of the participants was to examine a box of small wooden sticks to have the right size by inserting the sticks into a test device. This task was chosen after consultation with the supervisors of the SW. Since this is one of few tasks that is performed all year in the SW, most people working there were able to accomplish it. Therefore, the number of possible participants could be maximized using this task. Moreover, it can easily be transformed into a common hand over task, which is well-suited for the research performed. A bin with a small hole in the lid was used as a test device (compare Figures 1, 3). The wooden sticks had a length of ~70 mm and a width of ~5 mm. However, the width varied and sticks with a wider width had to be sorted out by the participants. This was done by inserting them into the hole in the test device. Here, sticks which were too wide would not fit through. On the first 2 days, where no robotic assistance was provided, the participants had a large stock of wooden sticks which they could access and test at their own working speed.

On the third day, during the collaboration with the robot, the robot picked up the wooden sticks on its table and then handed them over to the worker above their desk. The robot always approached the worker from the left side, using the same, linear trajectory, and same velocity. The time interval in which the wooden sticks were checked was thus defined by the robot.

In either execution, the test device was placed in front of the participant, who was free to place it where they felt most comfortable.

### Time Sequence of Experiment

In the run-up to the study, contact had been established with the heads of the SW in Oldenburg. In consultation with them, a suitable location and task was selected for the study. In addition, the participants and, where applicable, their legal guardians were provided with the participant information and declaration of consent in advance. At the beginning of the study, the participant information was handed out to the participants and their informal consent was again obtained. In order to prevent misunderstandings, the researcher was supported in doing so by the group leaders of the SW. Moreover, the study procedure was again verbally explained to them.

The two cameras and the scaffolding were already installed 1 week prior to the first day of the study, in order to familiarize the participants with the study situation and, so far, unfamiliar setup. The study itself took place on 3 consecutive days. On each new test day, the daily tasks were verbally explained once again. All participants took part in turn, and the order stayed the same for all 3 days. On the first day, the participant was only filmed by the two cameras while performing the task described above, and without any kind of attendance of the robot arm. The task was performed for about 30 min. This trial was intended to serve as a baseline condition. On the second day, the participants executed the same task. This time, the robot arm was physically present, but was turned off, standing sideways in front of the participant. The duration of the task execution decreased to 20 min per participant due to perceived repetition and according expressions of boredom relative to the first day. This round should serve two purposes. On the one hand, the familiarization with the test situation should be detected. On the other hand, in case of severe reactions to the robot, the further execution of the study would need to be adapted. The researcher was sitting directly opposite the participants on both days while writing the records of result.

On the third day, the robot arm performed the task of handing over the wooden sticks in front of the participants. The participants were instructed to remove the wooden sticks as soon as the robot stopped. The robot arm was positioned at five different distances from the participant. A maximum distance of 50 cm was chosen so that the participant could reach the chunks of wood without having to stand up. According to DIN 33402, 50% of all women have an arm length of more than 69 cm; with men this value is slightly higher. However, since the normal sitting position might be some centimeters apart from the desk, a somewhat smaller distance of 50 cm was chosen instead. In addition, a minimum distance of 10 cm was set in order to avoid an unintentional collision with the upper body of the participant. The intermediate distances are examined in steps of 10 cm. This means, a total of five different distances per participant were studied, which are 10, 20, 30, 40, and 50 cm anterior distance. Each distance measurement was performed five times in succession per participant. The order of execution of the different distances was randomized by counterbalancing it over the participants. In this trial, the additional third camera was used to record the participants from the front while the robot arm covered the participants in the original front camera. The researcher was next to the robot, wrote a record of the results and was able to assist with problems or questions at any time. Figure 3 shows a snapshot of a recording of this round.

**Figure 4:**
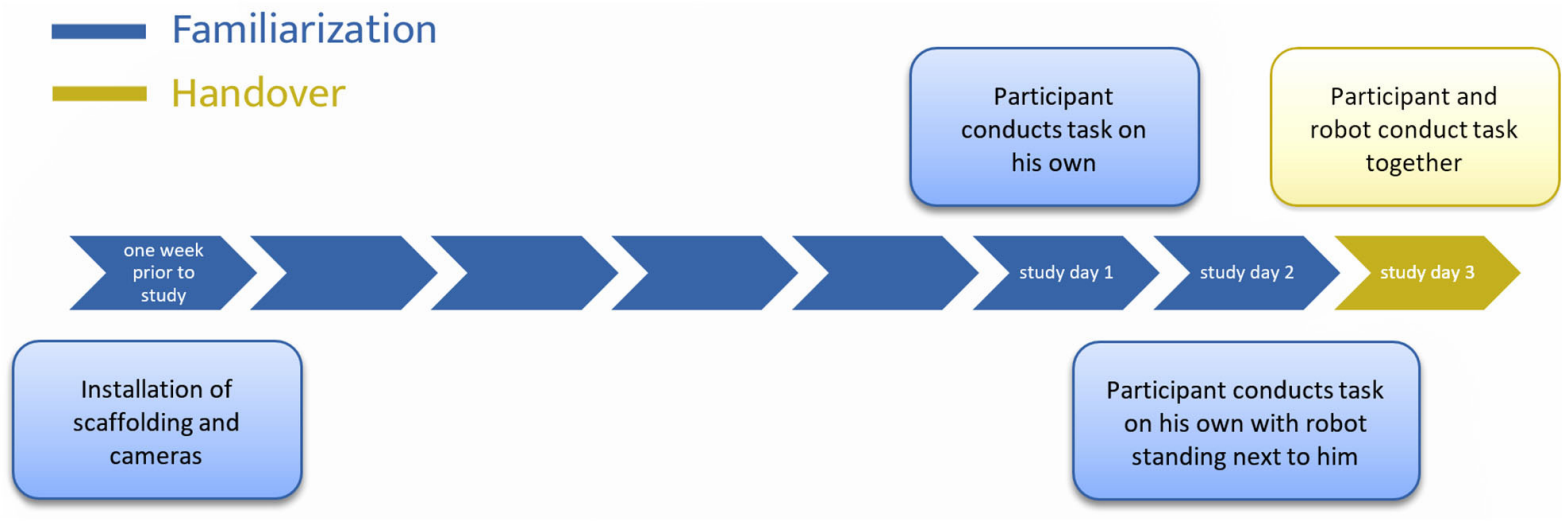
Timeline of the experiment.

Spread over the three test days, the study lasted for about 2 h per participant. All results were recorded via the cameras and the written record of the researcher. A survey questionnaire on the distance preferences of the participants was not carried out since most participants suffered from some kind of mental impairment. However, the supervisors of the work shelter later received mainly positive feedback from a small sample of the participants. The feedback varied from being very enthusiastic and happy to work with the robot arm to being rather bored because the robot arm did not move fast enough. No one gave feedback indicating fear or discomfort regarding the collaboration with the robot arm. Figure 4 once again visualizes the complete timeline of the experiment.

### Evaluation Metrics

The evaluation was conducted using a qualitative method and is similar to the single-case experimental design (SCED). Other than statistical methods in which groups are compared with each other, the participants in the SCED represent their own control group. The data is therefore compared within-subjects, rather than between-subjects. Usually, the comparison takes place between different time periods, in which a collected baseline phase is compared with subsequent phases (Smith, 2012). This method was chosen because acquiring a large number of participants with disabilities is infeasible within a single SW. Moreover, the variety in the participants' capabilities can vary strongly even when trying to acquire a homogenous group. This makes drawing statistical conclusions across all participants challenging. Nevertheless, datasets containing numerical values were tested for their significance, and are presented below.

The qualitative evaluation is based on the interpretation of certain, pre-defined variables, indicating some form of excitement, discomfort, or adaptation. Therefore, they will provide information about the overall acceptance of the robot. Since each participant will show individual reactions toward the new situation, the variables will be evaluated for every participant individually. All variables are either found in the record of results or the recorded videos.

#### Evaluation Parameter Familiarization

The process in which the participants got accustomed to being in a study situation and filmed by the cameras is defined as the familiarization phase. The initial introduction to the robot arm is another aspect that was investigated in the course of the evaluation. The familiarization process started 1 week prior to the first week of the study, when the cameras and scaffolding were installed.

Three variables were examined for both days: the overall body language, the speaking and the number of wooden sticks tested per minute. Additionally, on the second day, the reaction toward the robot was considered in terms of body and verbal language. To identify the familiarization process, these variables were proven with respect to changes in the behavior of the participants over time. Therefore, the time it took until a positive or negative change in behavior was detected was also considered for all variables and participants.

**Figure 5:**
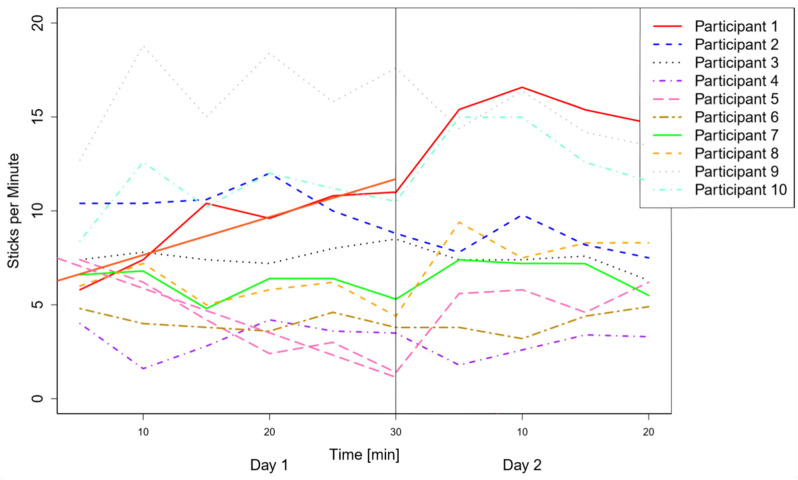
Sticks tested per minute for each participants and both days. Significant regression lines are included as well, which were found for participant 1 and participant 2.

The body language, the first variable, showed excitement and discomfort in a variety of ways. While some participants started out restless and hectic, others worked very quickly at the beginning and later on more calmly. A calming of either form can therefore be considered as a successful familiarization. For the second variable, the amount that the participant spoke was monitored; some did not dare to speak at all during the first day, while others started by fooling around. Therefore, again, any change in behavior is considered as a familiarization. The third variable, the amount of wooden sticks tested per minute, is intended to give an estimate about the overall performance, which is considered as another indication of well-being and excitement. While some participants started working faster due to excitement, others were distracted by the study situation and started more slowly. Thus, a change of the working speed in both directions can be considered as a sign of familiarization. The change was measured in terms of a significant linear regression in either direction on the 2 study days, as well as a comparison between both obtained regression lines per participant. The significances were tested using a *t*-test and a F-statistic. This can be seen in Figure 5. It should be noted that, for seven participants, the task execution was terminated 1–2 min before the actual end of the trial period for different reasons, such as attention deficit or an earlier start to their break. However, these kinds of occurrences are unavoidable and a repetition of the measurement unreasonable in this target group. Therefore, the missing minutes were extrapolated for all participants in order to obtain a comparable dataset, except for participant 5 who had no missing data.

Above variables were compared for both days, pointing toward changes in behavior and a successful familiarization. For the second day, a fourth variable, the reaction toward the robot arm was examined. With this variable, negative reactions such as turning away one's body, being distracted by looking at the robot arm or avoiding its close approach were considered, as well as whether a change toward a more positive attitude occurs. All four variables, for both days, can be seen in Table 2.

**Table 2:** Four variables indicating some form of excitement or discomfort are described for each participant.

**Participant**	**Body language**			**Speaking**			**Sticks per minute**	**Reaction toward robot**
	**Change**	**Duration**	**Symptom**	**Change**	**Duration**	**Symptom**	**Significant regression**	**Change**
1	Yes	22 min	Restless, replacing box	Yes	Complete day 1	Does not speak	Yes	No
2	No	–	–	Yes	Complete day 1	Does not speak	No	No
3	Yes	20 min	Concentrated, hectic	Yes	Complete day 1	Does not speak	No	No
4	No	–	–	No	–	(Never speaks)/Speaks	No	No
5	Yes	16 min	Awake, fast	Yes	20 min	Does not speak	Yes	No
6	Yes	23 min	Hectic stick placing	No	–	(Never speaks)/Speaks	No	No
7	No	–	–	Yes	16 min	Does not speak	No	No
8	No	–	–	Yes	17 min	Fooling around	No	No
9	Yes	20 min	Works very fast	Yes	15 min	Does not speak	No	No
10	No	–	–	No	–	(Never speaks)/Speaks	No	No

“*Change” states whether a change from the normal behavior was detected, “duration” states how long the abnormal behavior lasted and “symptom” describes how this behavior showed*.

#### Evaluation Parameter Handover

**Figure 6:**
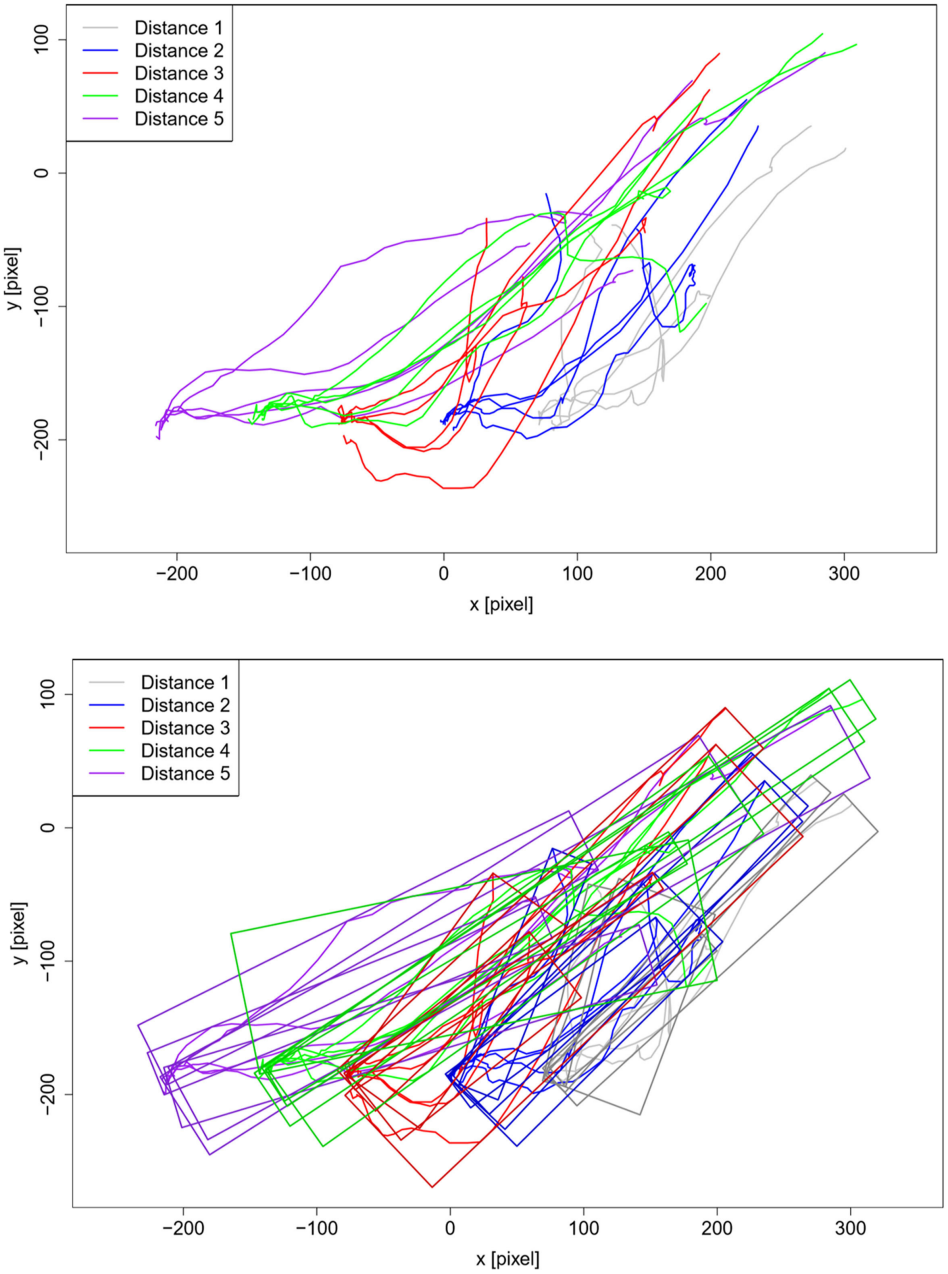
**(Top)** Trajectories for all five distances extracted from the videos for participant 1. The *x*- and *y*-coordinates of the participants hand are plotted for each timestep in the two-dimensional space. The start position of the participants hand is on the upper right corner, the stop positions of the gripper are at the bottom end of the individual curves. **(Bottom)** Same as top, but with additional oriented bounding boxes for all five trajectories per distance. 0 indicates the origin of the image.

The same procedure as for the familiarization phase was used to evaluate the reaction to collaboration with the robot arm in general, as well as at different distances on the third day. Five variables were considered to be relevant (see top row of Table 3). Other than during the familiarization process, not a change in variables is considered here, but the overall perceived negative or positive feelings. The first variable is a change in posture during the grasp and while changing the robot distance to indicate a comfortable or uncomfortable grasping position, and the first reaction toward the new distance. If the participant had to lean forward to grasp the stick, or to reposition the chair to interact with the robot at a new distance, they were assumed to be uncomfortable. The second variable looks at negative reactions for the participant toward the robot arm in general, such as turning away their body, being distracted by looking at the robot arm or flinching when the robot arm and participant approach each other. Positive reactions, such as smiling toward the robot arm were considered for the third variable. These variables were all taken from the records of results. Additionally, the trajectories of the participants' hands during grasping (i.e., the approach to the robot) were extracted from the video, using the software “Kinovea” (France) (Kinovea, 2019). The resulting *x*- and *y*-coordinates of the participants' hand movements are plotted for each timestep in the two-dimensional space. Based on these trajectories, the minimal oriented bounding boxes (OBBs) of the paths were calculated, in which a small width of the OBB, i.e., small deviation from a linear path, is considered to be a sign of a comfortable movement toward the robot. A large OBB is associated with possible evasion movements or hesitation. The size of the OBB is thus investigated for the fourth variable. For the extracted trajectories, an accuracy of 2 pixels in either direction is assumed, leading to an accuracy of 4 pixels of the OBB when applying error propagation. Here, one centimeter equates to ~6 pixels. Figure 6 shows the obtained trajectories and corresponding OBBs for one exemplary participant. The trajectories and OBBs for the other participants can be seen in the Supplementary Figures 1–8. Participant 8 was excluded from this evaluation since he has a visual impairment. The obtained trajectories are thus rather a result of search movements than of actual reactions toward the robot.

**Table 3:** Five variables indicating a reaction toward the robot and its distance for each participant.

**Participant**	**Distances of change in** **posture (1–5)**		**Negative reactions**	**Positive reactions**		**Oriented bounding box width of arm trajectories**	**Time difference between start human grasp and stop robot gripper**
	**During interaction**	**While changing robot distance**	**Reaction**	**Reaction**	**Indication**	**Distance (1–5) ranking from smallest to widest box [identical within error margin of 4 pixel]**	**Distance (1–5) ranking from fastest to slowest reaction [identical within error margin of 0.26 s]**
1	4, 5	–	No	Yes	Smiling toward robot	1, 2, 5, [3, 4]	3, [4, 2, 1], 5
2	4, 5	1	No	No	–	1, 5, [2, 3], 4	–
3	5	1	No	Yes	Smiling toward robot	[4, 2], 5	[2, 4, 5]
4	5	–	No	Yes	Smiling toward robot	[4, 2, 1], [5, 3]	3, [*4, [1, 5*], 2*]*
5	1, 2, 4, 5	1, 2, 4, 5	No	No	–	[*2, [3*], [*5], 1*], 4	1, 2, 5, [4, 3]
6	5	1	No	No	–	[2, 5, 3, 1]	[*5, [3*], 2, 1*]*
7	4, 5	1, 2	No	No	–	1, [3, 2], 4, 5	[5, 2], 1, 4, 3
8	4, 5	2	No	No	–	–	–
9	4, 5	–	No	Yes	Smiling toward robot	1, 2, [5, 3, 4]	[2, 1], [*5, [3*], 4*]*
10	5	–	No	Yes	Smiling toward robot	5, 4, 2, 1, 3	[*1, [3*], 2, 4, 5*]*

*The findings with respect to distance are represented with 1 meaning 10 cm distance and 5 meaning 50 cm distance between robot and participant. For the last two variables, a ranking is presented with distances having the same result within the error margin being displayed in brackets*.

The fifth variable considered is the time difference between the robot arm stopping and the participants' grasping movement starting. A fast reaction, especially a movement while the robot arm is still moving, is associated with less fear. The accuracy of the extracted times is considered to vary by up to two frames, which corresponds to 0.13 s in the video recording. After propagation of uncertainty, a total error margin of 0.26 s is assumed for the time difference. For this evaluation participant 8 was again excluded because the robot arm did not make sufficient noise to be heard in its current state. Additionally, participant 2 was excluded due to a short-term memory loss, which led to the participant forgetting the task after each trial. Both participants thus only grasped the stick on request and no information on confidence could be extracted. The data collected for variables 4 and 5 were initially tested for a significant correlation regarding the distance of the robot and reactions of the participants associated with these variables. A preliminary Shapiro-Wilk test revealed for both cases that the data is not normally distributed. For this reason, a subsequent Friedman test was applied, which showed no significance for either variable. For this reason, a qualitative analysis was performed instead, in alignment with the other variables. All results are listed in Table 3. In addition, the single values of variable 4 and 5 can be seen in the Supplementary Tables 1, 2. As in the previous section, some data points are missing; these were excluded from the evaluation for the reasons detailed previously.

## Results

The results are presented in two separate sections; the first will cover the process of becoming familiar with the study situation and the robot arm itself, while the second section will cover the actual interaction with the robot arm at different distances.

### Familiarization

The installation of the cameras and scaffolding did not cause any specific reactions, as reported by the group supervisors.

Eight out of 10 participants showed at least one kind of reaction according to the predefined variables and a resulting change in behavior (see Table 2).

The first variable, the body language, served for five out of 10 participants as evidence of excitement, with the unusual behavior lasting, on average, for 20.2 (±2.4) minutes during the first day. No significant changes in this variable were noticed during the second day for any of the participants. The amount of verbal interaction, the second variable, revealed signs of excitement in 7 out of 10 participants. Four of those showed a relaxed mentality after 15–20 min, while no improvement could be witnessed during the first day for three participants. During the second day, all participants displayed their typical way of interacting with the environment in terms of talking and laughing, as confirmed by the group supervisors. The third variable, the number of sticks tested per minute, only showed significant changes for two out of nine participants on the first day. The elapsed time significantly predicted the number of sticks tested per minute for participant 1 [*b* = 0.20, *t*_(4)_ = 4.09, *p* < 0.05] and participant 5 [*b* = −0.24, *t*_(4)_ = −6.65, *p* < 0.01], with *b* representing the slope of the line, *t* being the *t*-value and *p* describing the *p*-value of the independent variable. The overall model with the elapsed time also predicted the number of sticks tested per minute very well [adjusted *R*^2^ = 0.76, *F*_(1,4)_ = 16.69, *p* < 0.05] and [adjusted *R*^2^ = 0.90, *F*_(1,4)_ = 44.22, *p* < 0.01], respectively. Here, adjusted *R*^2^ represents the effect size, *F* describes the F-statistic depending on the number of dependent variables and the degrees of freedom and *p* being the *p*-value of the model. On the second day, no significant changes could be detected regarding the number of sticks tested per minute for any of the participants. Moreover, none of the participants showed any kind of negative reaction toward the robot arm during the second day, as the evaluation of the last variable revealed.

### Handover

The first variable, the change in posture during the grasp and while changing the robots distance, showed that all 10 participants had to lean forward to reach position 5 and six participants still had to lean forward to reach position 4 (see Table 3). In addition to this, five participants flinched while the robot was moved to distance 1 and three while it was moved to distance 2. However, all except participant 5, who was sitting in a wheelchair, approached the robot again to continue the trial after the alteration of distance was completed.

When considering the second variable, negative reactions toward the robot, it becomes apparent that none of the participants showed any signs that could be interpreted as fear or discomfort toward the robot arm. Instead, five out of 10 participants showed signs of joy during their first interaction, as the evaluation of the third variable revealed.

**Figure 7:**
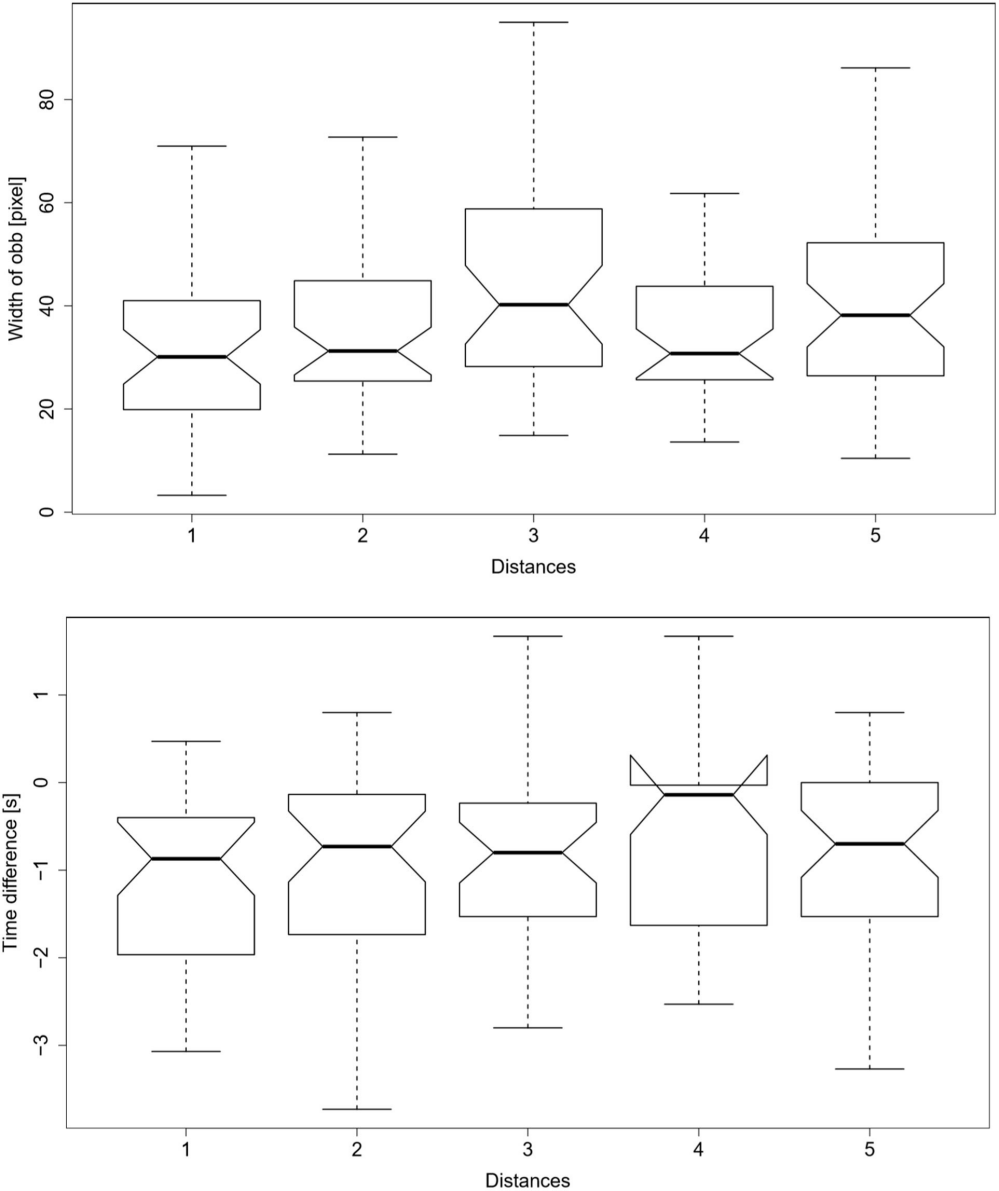
Boxplots of the oriented bounding boxes of the trajectories of the participants **(Top)** and time differences between the stopping of the gripper and the start of the participants' grasping movement **(Bottom)** over all participants and distances. Distance 4 reveals the absence of data points. Note that a negative time difference represents that the grasp motion of the person starts before the robot finishes its motion.

The fourth variable, the size of the OBB, showed for five out of nine participants the smallest OBB for distances 1 and 2, while only for one it was smallest for distance 5. The remaining three participants did not show preferences considering the error margins. Similar is true for the time differences, the fifth variable. Three out of eight participants reacted fastest for distances 1 and 2, while only one reacted fastest for distance 5. The remaining four participants either showed the fastest reaction for distance 3 or no preferences could be estimated considering the error margins. The mentioned tendencies for variable 4 and 5 can be seen in Figure 7.

Also of note is a single finding in which a person, performing a kind of tic, namely spinning the wooden sticks in his hands for several seconds before placing it into the box, actually abandoned this tic when the robot arm handed over the wooden sticks. In general, the feedback the supervisors received from participants varied from being very enthusiastic and happy to work with the robot arm to being rather bored because the robot arm did not move fast enough. No one gave feedback indicating fear or discomfort regarding the collaboration with the robot arm.

## Discussion

The following sections will give a brief overview of the findings and conclusions of this work, as well as of its limitations and suggestions for future work.

When discussing the results, it must be emphasized that the study is based on a small and diverse sample. Therefore, the transferability of our conclusions to other participants and other experimental settings could be limited. Given the target group, the small size, heterogeneity, and gender-unbalance in the sample is inevitable. The SW is organized into working groups. Each of these groups consists of people with varying types and levels of disability, different ages and gender. The study was conducted in one specific working group but in order to include as many participants as possible, all people with disabilities working in the SW qualified to take part in the study. The only requirement was to have the capability to perform the task at hand in order to prevent additional workload and to yield a small form of homogenization among the participants. These minor requirements, together with the participants' willingness to participate in the study, led to the acquisition of only 10 participants out of a total of 300 employees. A further limitation in terms of gender and type of disability was therefore unfeasible. Moreover, as described in the Introduction, it is very difficult to produce homogeneity within the special target group, particularly since SWs include people with varying types and degrees of disability. The heterogeneity of the group also led to the fact that two participants had to be excluded from the evaluation of some variables. One participant was blind and the other had short-term memory loss. Therefore, for example, the time until the grasp took place could not be measured for these participants, and the underlying data used for the evaluation had to be reduced to eight participants. Nevertheless, since the exclusion of the participants from the whole study would lead to a reduced number of participants for all variables, as no additional participants could be recruited, the decision was made to include both. The possibility that participants would suddenly be excluded from the study is something that has to be kept in mind for future research, as it is a common occurrence for this type of target group. Due to the listed limitations, the conclusions drawn in this paper are suggestive and do not reflect statistically significant results. However, as outlined by Lindblom et al. (2020), qualitative evaluations are not necessarily of less scientific rigor, but are used for different aims compared to quantitative studies. They are especially useful for early testing stages to identify the cause of the triggered action and how to fix it (Lindblom et al., 2020).

The results of the first 2 days suggest that a familiarization with the study situation and an industrial robotic arm can be achieved in a relatively small amount of time. The strongest feelings of nervousness subside after only about 20 min for most participants. None of the participants showed any ongoing signs of aversion to the study situation and the robot arm did not cause any negative reactions for any of the participants. These findings contrast with assumptions in literature that people with disabilities adapt slowly to changes and are more prone to react strongly to outer stimuli (Clark, 2000; Kremer et al., 2018). Furthermore, on the third day, when collaborating with the robot arm, none of the participants showed any signs of fear or discomfort. Therefore, it can be assumed the new technology would be widely accepted. This might be due to the fact that people with disabilities rely more often on assistance and help from technology and thus accept this kind of help more easily.

Surprisingly, when considering the different distances, it became apparent that shorter distances are not inferior to longer distances, as initially thought. The short distances did not cause the participants to show any signs of distrust toward the robot but tendentially led to faster execution, shorter taken paths, and less inconvenient postures. Moreover, when flinching of a participant was observed, it took place while the researcher altered the robot distance and not while the robot moved by itself. All participants except for one approached the robot again after the researcher had finished repositioning the robot. Also worth mentioning is that the participant who did not reapproach after the alteration was completed, was the only person sitting in a wheelchair. Thus, this behavior might be due to convenience rather than fear. These observations led to the conclusion of the distrust being rather directed toward the researcher than toward the robot arm. Consequently, the trust toward the robot arm might be larger than toward humans. This would be in line with the aforementioned studies (Giullian et al., 2010; Ricks and Colton, 2010; Jouaiti and Hénaff, 2019; Reardon et al., 2019) about autistic children and students becoming socialized and educated more easily with robots than with humans. Also, the single finding that one participant abandoned his tic lends weight to this hypothesis.

## Conclusion

The paper at hand presents a first field study toward the acceptance of an industrial robot arm in a SW by people with disabilities. In order to separate any effects that are not caused by the moving robot, first the process of familiarization with the study situation and technical equipment was considered and examined. Second, the reaction toward the robot arm during collaboration was investigated. Here, different distances between the robot arm and human were considered to include the effects of social distances into the results.

Three main findings were achieved. Firstly, a familiarization can be achieved in a limited time. Secondly, a collaboration between adult humans with disabilities and an industrial robot is accepted more readily than has been assumed in literature so far (Kremer et al., 2018). Finally, a close positioning of the robot arm does not cause a problem, as long as safety aspects can be guaranteed.

According to the findings, a familiarization phase is useful and should last for about 20 min in order to get consistent results in the subsequent study. Due to the rather short span of attention of some people with disabilities, a familiarization phase 1 day prior to the study is recommended. Additionally, a better guarantee of familiarization can be achieved that way since the measured speech of some participants took 1 day to return to normal.

Overall, the results suggest that the use of a robotic arm is a promising field of research. People with disabilities seem to be willing to use technology in order to master tasks at work and none of the participants showed any signs of fear or discomfort.

Not even a spatially close collaboration with the robot arm showed any negative effects. In contrast, the shorter distances appeared to be slightly preferred over the longer ones. Positioning the robot too far away led to uncomfortable postures during execution of the task. Subsequently, as long as safety requirements can be satisfied, there seems to be no limit regarding a minimal distance.

This paper presents an important first step toward human robot interaction with people with disabilities, as we now know that no negative impact results. This invites future scenarios in which a robot could help people with disabilities to be integrated into the open labor market more easily.

### Limitations and Future Work

The primary limitation of this study is the small group size. Furthermore, the homogeneity was achieved based on their smallest common thread, the capability to execute the given task. Since no restrictions based on kind and severity of the disability were made, still a large inhomogeneity of the capabilities of the participants can be assumed. Also, the effects of gender and type of disability could not be investigated in a statistically significant way, due to the limited source of participants. This means, further studies with more participants would be desirable in order to obtain statistical results, although they are most likely unfeasible.

In this study, only one parameter was changed, namely the distance to the robot arm, however, other factors such as velocity, noise, trajectory, and many more might have an influence on the acceptance. The working speed, for example, was prescribed by the robot, and therefore no conclusion on whether the productivity can be increased can be drawn from the results. This, however, is an important point to evaluate other aspects of the acceptance like the perceived usefulness, as proposed by Davis (1989).

While further studies on these subjects would be desirable, covering them all would have exceeded the scope of this study. Nevertheless, the general acceptance of the robot arm was tested. Moreover, the main aim in employing workers with disabilities is usually a charitable one rather than the desire to maximize the productivity level. For this reason, the parameters can be adapted to suit the worker if needed, while the robot could still improve their working capabilities. However, this hypothesis should be tested in a subsequent study, as well as the aforementioned possibility that people with disabilities may trust robots more than humans and might therefore even learn better with them. If this hypothesis proves true, collaboration with a robot arm at the workplace might have even more benefits than expected prior to this study. However, this aspect would need further investigation to be validated.

In summary, proceeding with the initial visions for human-robot collaborations in SWs should be continued in future work.

## Data Availability Statement

The datasets generated for this study will not be made publicly available because they are based on personal video data of the participants and therefore cannot be publicly released due to data-protection law and ethical aspects. Requests to access the datasets should be directed to Sandra Drolshagen, sandra.drolshagen@offis.de.

## Ethics Statement

The studies involving human participants were reviewed and approved by Commission for Research Impact Assessment and Ethics, Carl von Ossietzky University Oldenburg, Germany. Written informed consent to participate in this study was provided by the participants or if required by the participants' legal guardian/next of a kin.

## Author Contributions

The main author is SD, who developed the study design, programmed the robot, and performed the study and evaluation. MP supported the project with his professional knowledge and also took part in the programming and development of the study design, as well as in inventing strategies for the evaluation. PG supported the project by helping to implement the required safety requirements. AH is the chief scientist, supporting the development of study design and strategies throughout the whole process. All authors contributed to the article and approved the submitted version.

## Conflict of Interest

The authors declare that the research was conducted in the absence of any commercial or financial relationships that could be construed as a potential conflict of interest.
