# Supplementary material for: Acceptance of Industrial Collaborative Robots by People With Disabilities in Sheltered Workshops
Source: Front Robot AI. 2021 Jan 11;7:541741. doi: 10.3389/frobt.2020.541741 (PMC7831153; doi:10.3389/frobt.2020.541741)
Supplement: Supplementary file 1 [file Data_Sheet_1.docx]

Supplementary Material

## Risk Analysis

Tabel 1: Risk analysis for the robot arm operating in the SW.

a) Declaration of key figures.

| Probability of occurrence | % | Consequence | Explanation |
| --- | --- | --- | --- |
| 5 | < 90 % | 5 | fatal |
| 4 | < 70 % | 4 | dangerous |
| 3 | < 40 % | 3 | undesirable |
| 2 | < 20 % | 2 | disturbing |
| 1 | < 5 % | 1 | negligible |

b) Estimated risks and action to minimise the risks.

| # | Risk | Cause | Consequence | Probability of occurrence | Consequence | Action to minimise the risks |
| --- | --- | --- | --- | --- | --- | --- |
| 1 | Crash of robots control system | Technical problems | The robot arm stops and does not carry out its activity. The participant could be irritated. | 2 | 5 | The researcher sits next to the robot and can correct the error. Before the study is carried out, the robot is inspected according to the manufacturer’s specifications.   - Consequence reduces to 2 |
| 2 | Free impact/transient contact with employees | Participant bends forward or stretches out his or her arm the moment the robot operates there. Other employees reach into the robot's workspace. | The participant or the employee gets injured. | 2 | 5 | The researcher holds an emergency stop switch in his hand to avoid a collision. Nevertheless, if such a case occurs, the maximum speed, force, and pressure have been limited to 50 % of the maximum permitted value in accordance with ISO TS 15066. The robot's working area is also limited in such a way that it can only move above the working desk but not freely in the room.   - Probability of occurrence is reduced to 1 and consequence reduces to 2 |
| 3 | Clamping/quasi-static contact with employees | Participant or employee has his hand on the depositing position the moment the robot moves there or between the gripper when it closes. | A body part of the participant or the other employee is injured or squeezed. | 1 | 5 | The researcher holds an emergency stop switch in his hand to avoid a collision. Nevertheless, if such a case occurs, the maximum speed, force, and pressure have been limited to 50 % of the maximum permitted value in accordance with ISO TS 15066.   - Consequence reduces to 2 |
| 4 | Detaching workpieces | The workpiece was not gripped correctly and is released from the gripper during the movement. | The gripped workpiece can fly around and hit the participant or an employee. | 1 | 4 | The workpiece is match-sized wood, which does not have a large weight and therefore, cannot cause severe injuries if it does not hit sensitive parts of the body, such as the eyes. However, by reducing the speed of the robot, no large centrifugal forces are build up here either.   - Consequence reduces to 1 |
| 5 | Anxiety attack | Fast, unexpected or loud movements and noises of the robot. | The participant gets scared. | 2 | 3 | The robot is stopped and a supervisor is called in.   - Consequence reduces to 2 |
| 6 | Trapped in the robot | Long, open hair or loose clothing of workers. | Injuries to the employee and torn clothing. | 2 | 4 | Employees and participants must have long hair tied and wear close-fitting clothing. If such contact does occur the maximum forces and pressure values set will stop the robot.   - Probability of occurrcence is reduced to 1 and consequence reduces to 2 |
| 7 | Sharp edges | Sharp edges at the end effector (the robot itself has no sharp edges). | Injuries of the participants or other employees. | 1 | 3 | The sharp edges of the end effector are wrapped with foam.   - Consequence reduces to 1 |
| 8 | Upsetting of the robot | Unintentional contact with the robot arm or its own movements will cause it to tip over. | Damage to the robot arm and injuries to nearby employees. | 4 | 4 | The robot arm is bolted to the table, which prevents it from falling over.   - Consequence reduces to 1 |

## Study Results


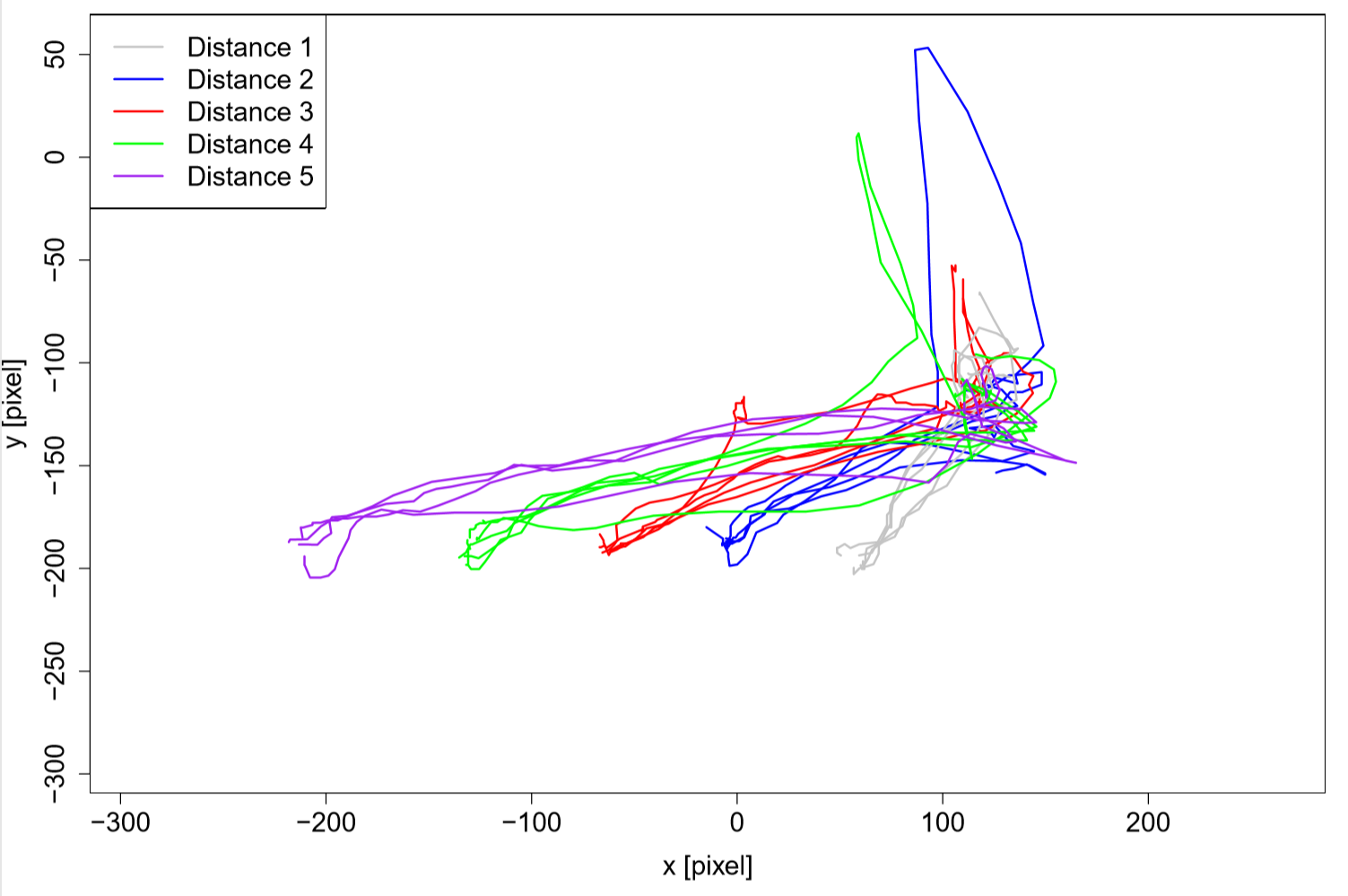

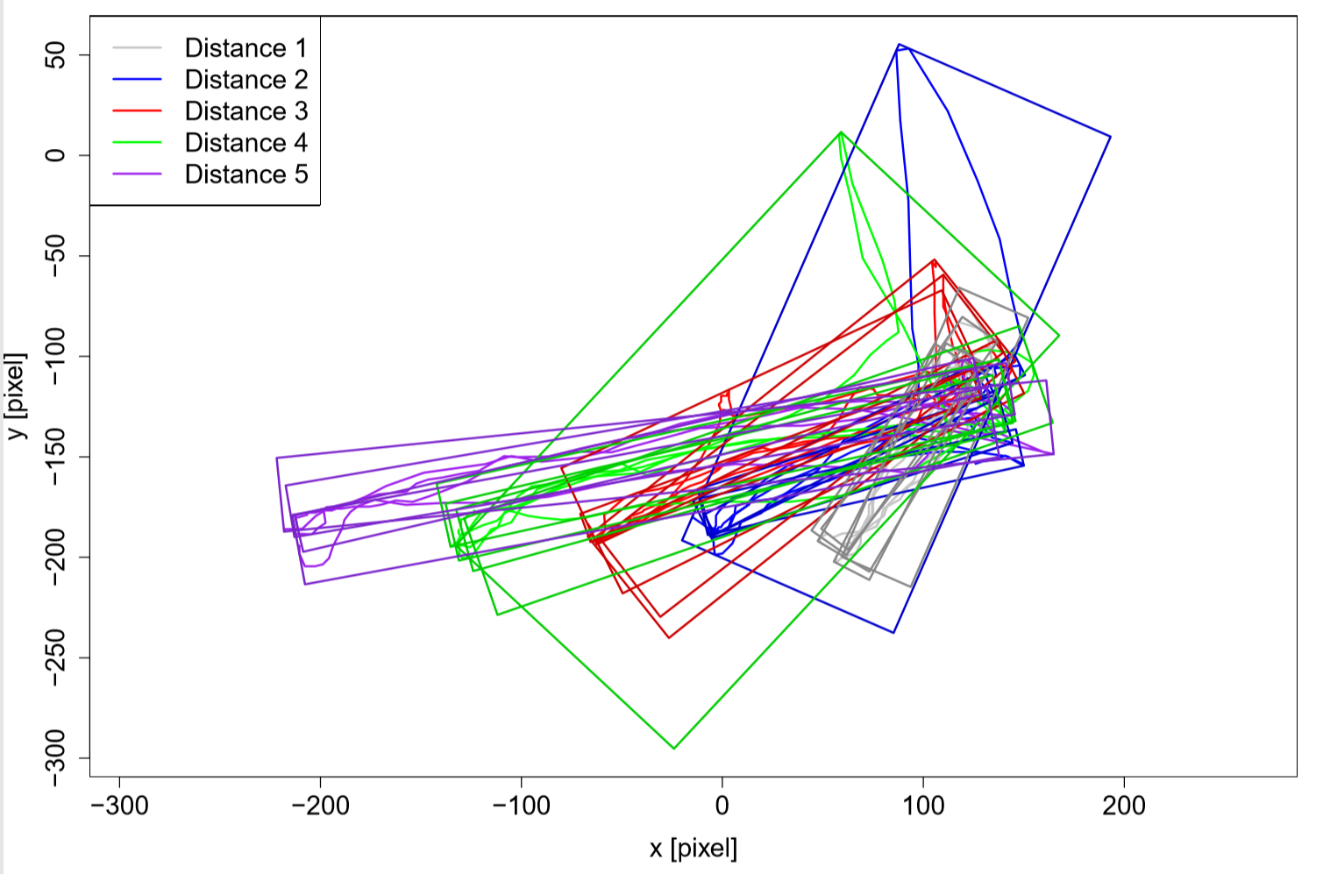


Figure 1: Top: trajectories for all five distances extracted from the videos for participant 2. The start position of the participants hand is on the upper right corner, the stop positions of the gripper at the bottom row. Bottom: additional oriented bounding boxes for every trajectory.


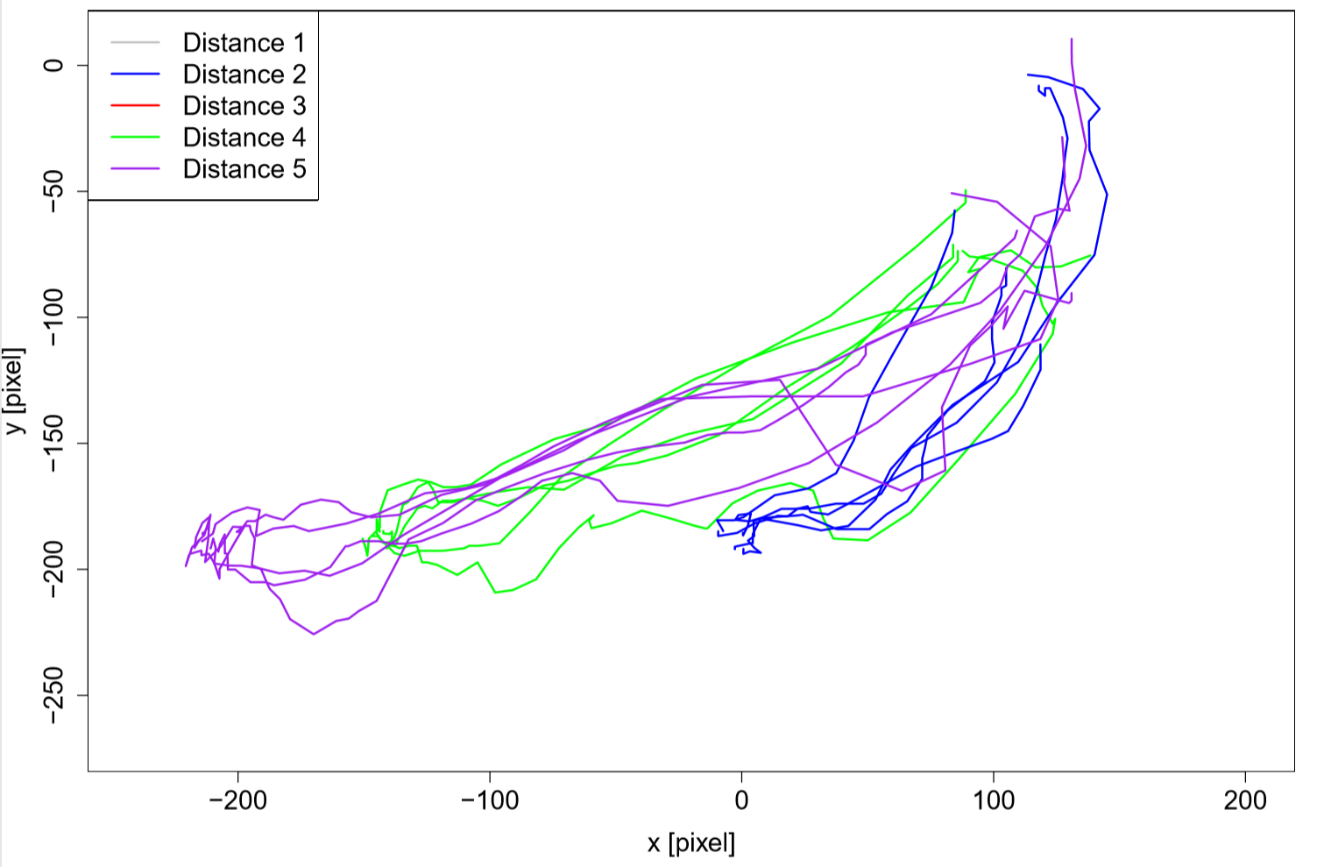

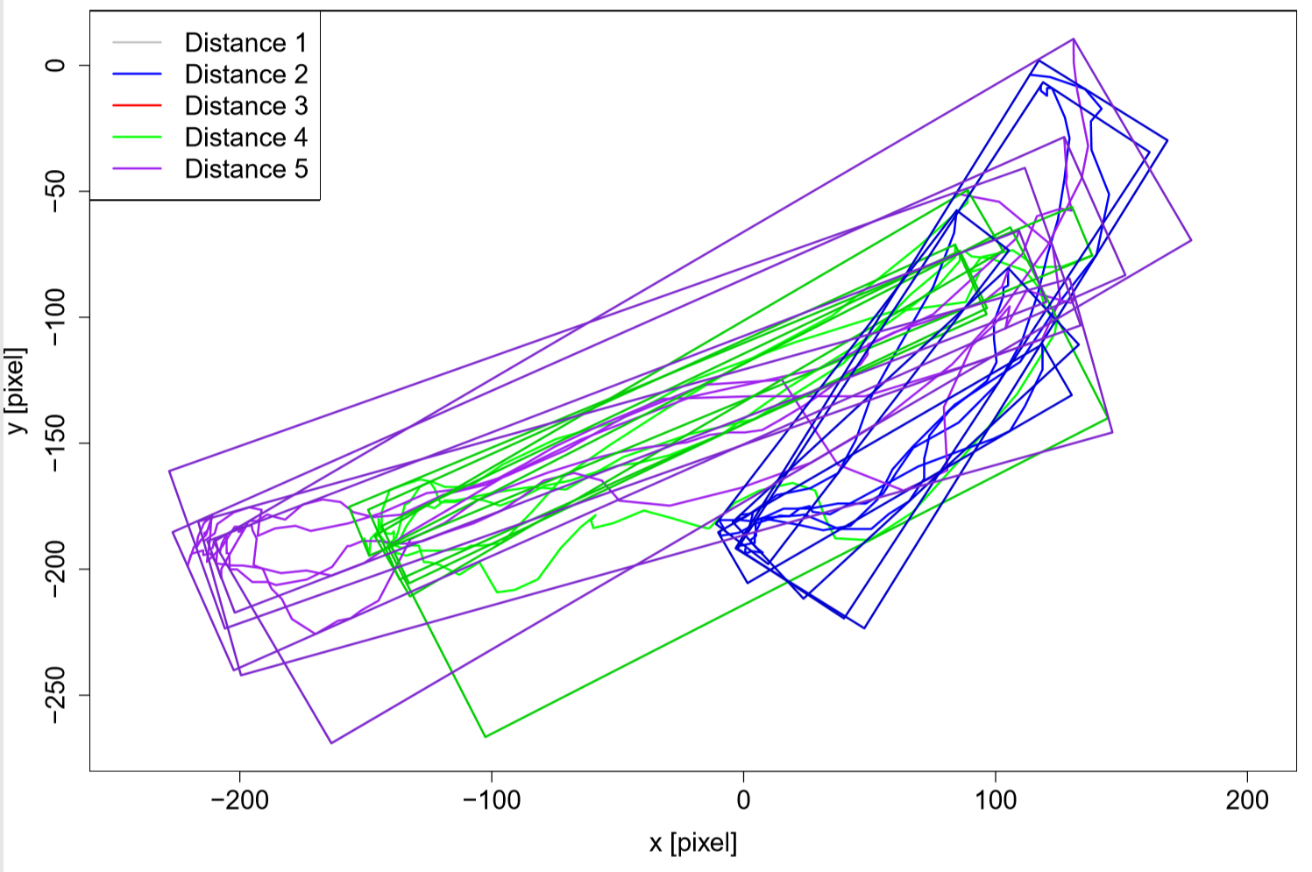


Figure 2: Top: trajectories for all five distances extracted from the videos for participant 3. The start position of the participants hand is on the upper right corner, the stop positions of the gripper at the bottom row. Bottom: additional oriented bounding boxes for every trajectory.


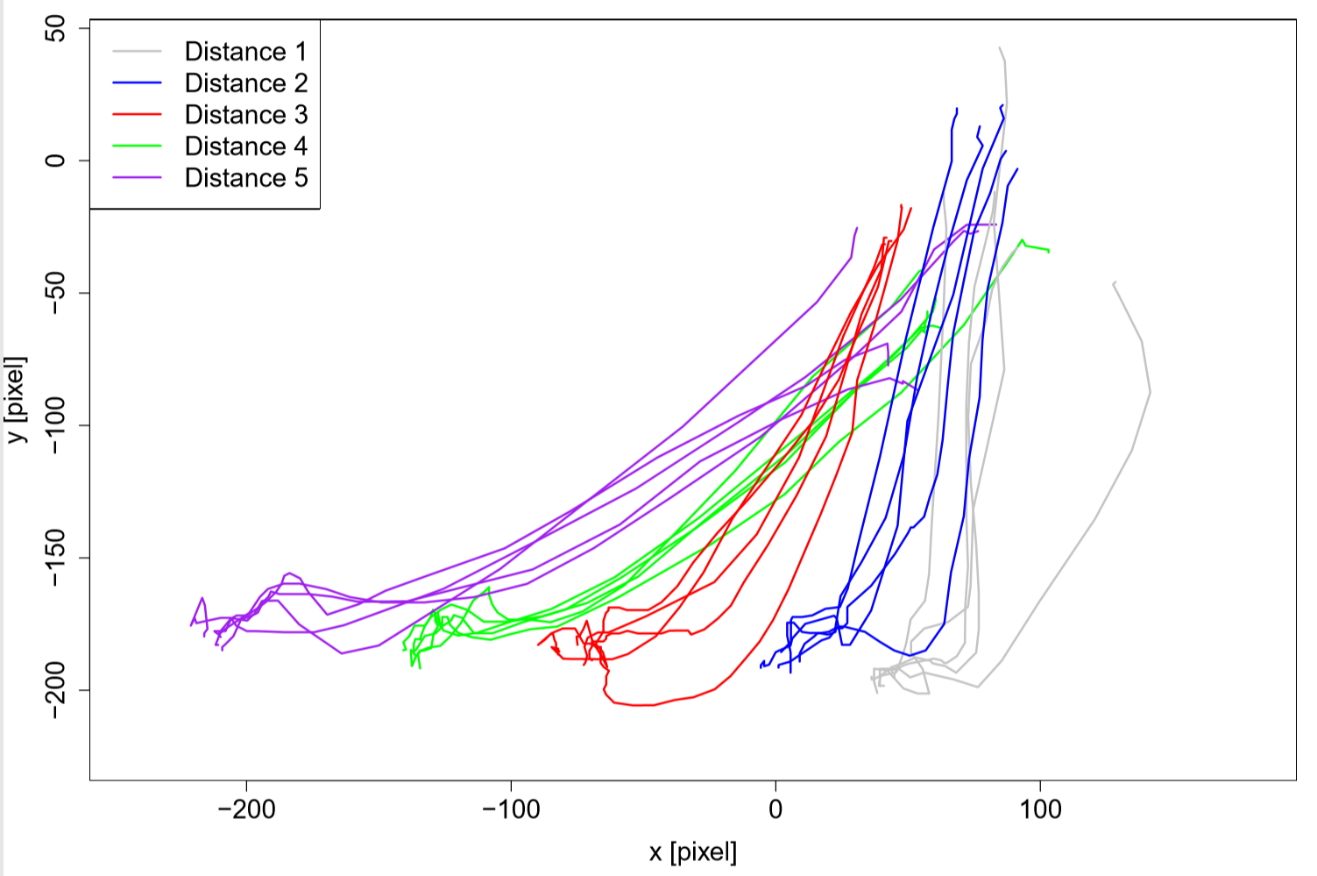

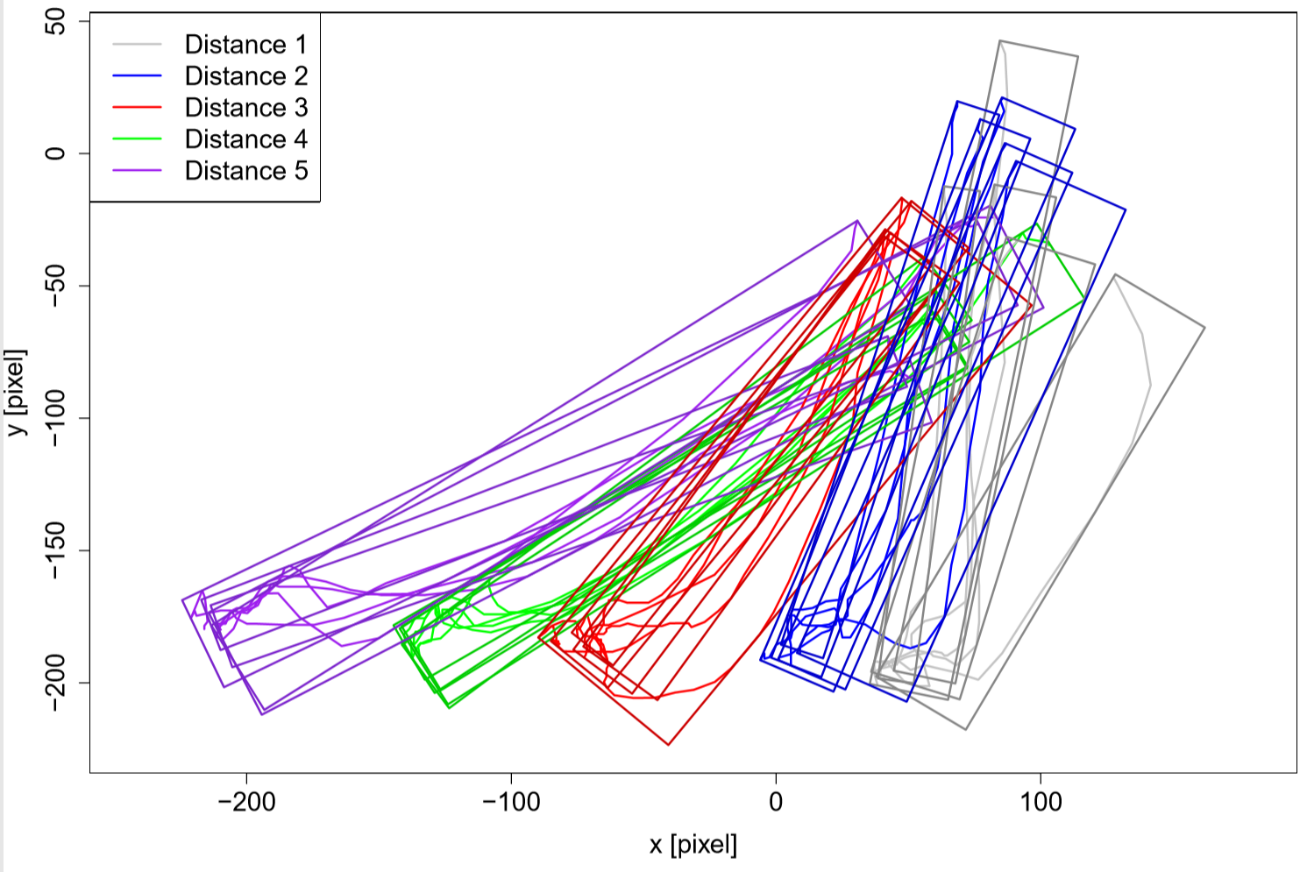


Figure 3: Top: trajectories for all five distances extracted from the videos for participant 4. The start position of the participants hand is on the upper right corner, the stop positions of the gripper at the bottom row. Bottom: additional oriented bounding boxes for every trajectory.


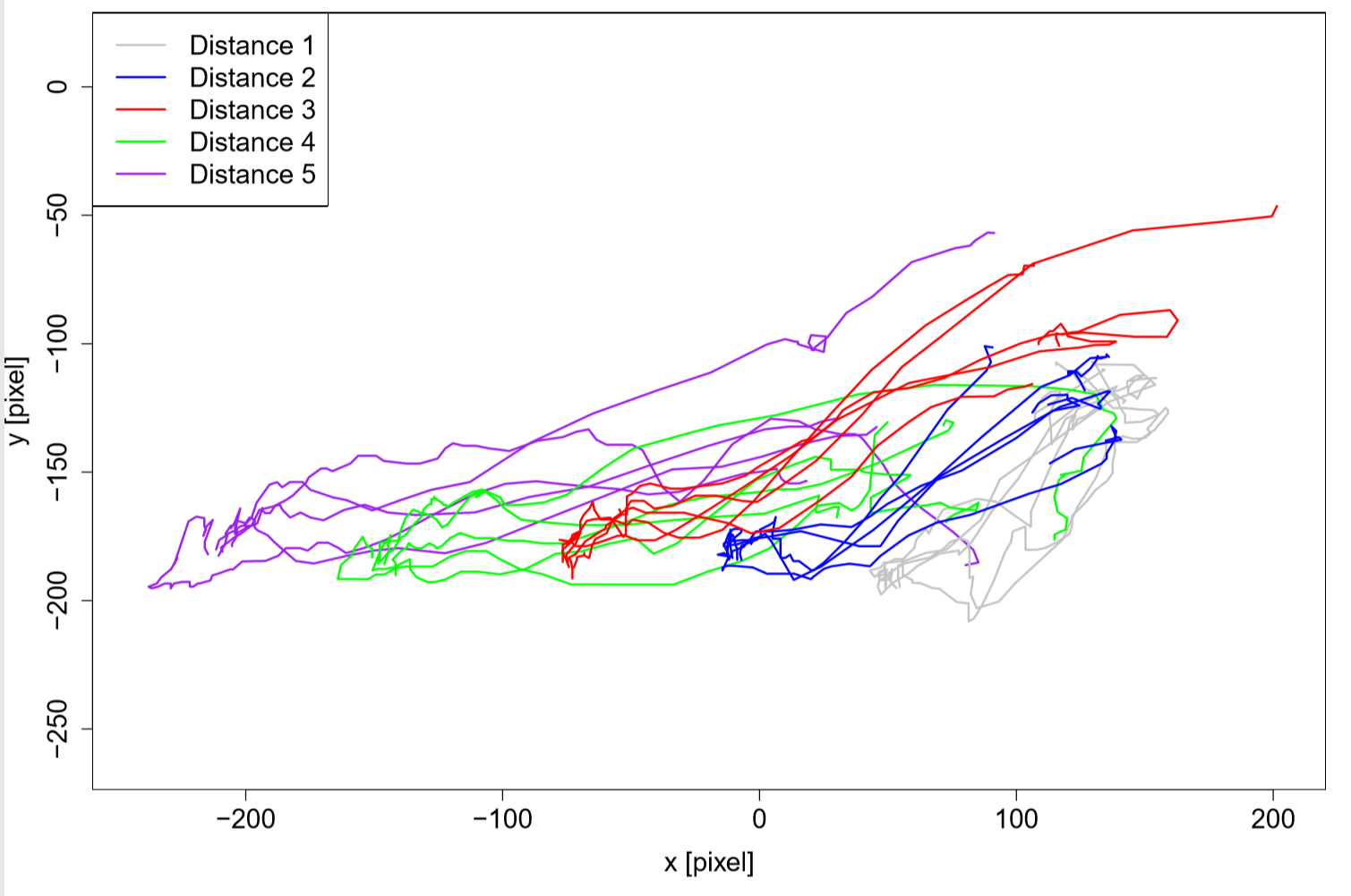

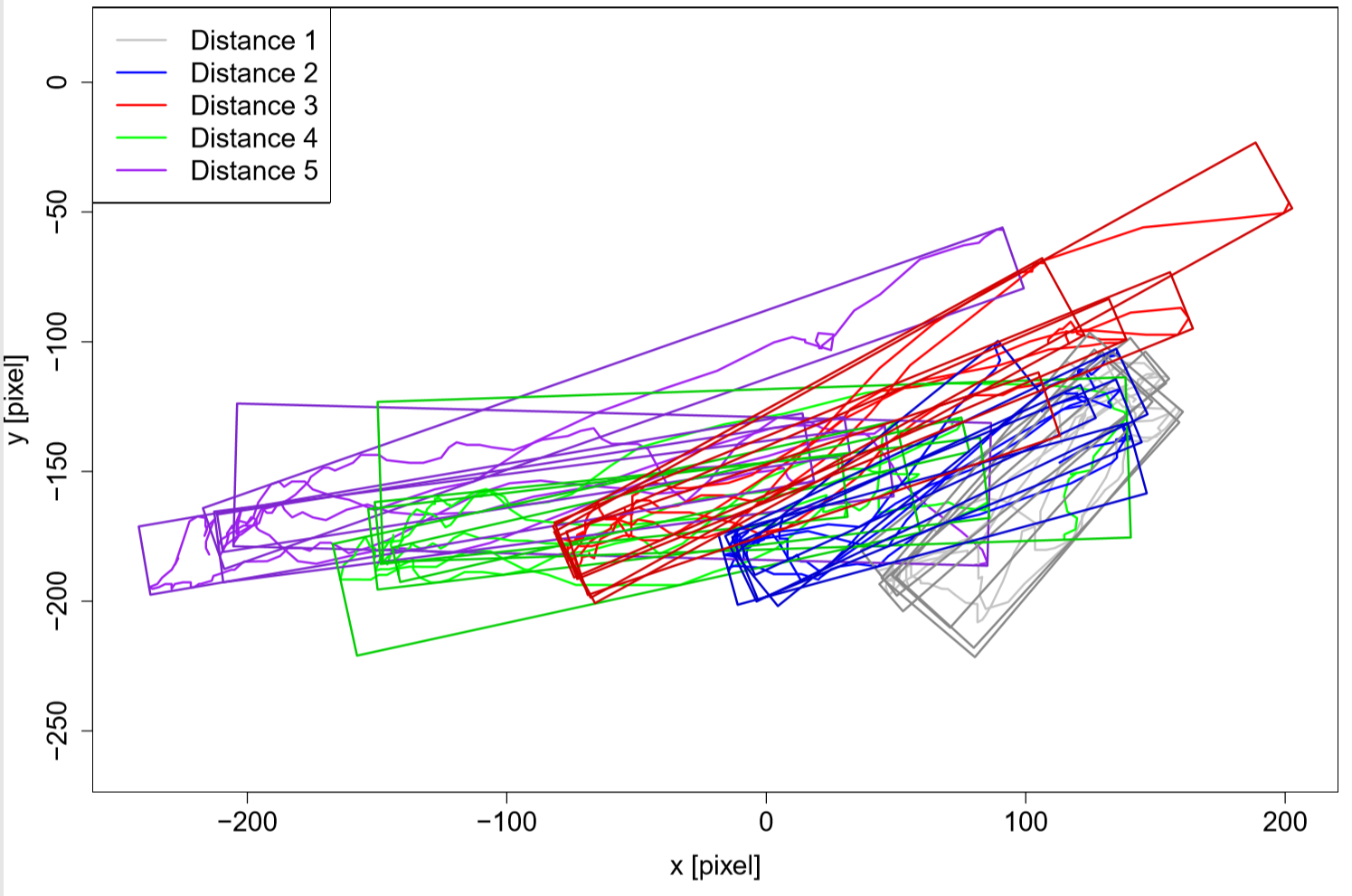


Figure 4: Top: trajectories for all five distances extracted from the videos for participant 5. The start position of the participants hand is on the upper right corner, the stop positions of the gripper at the bottom row. Bottom: additional oriented bounding boxes for every trajectory.


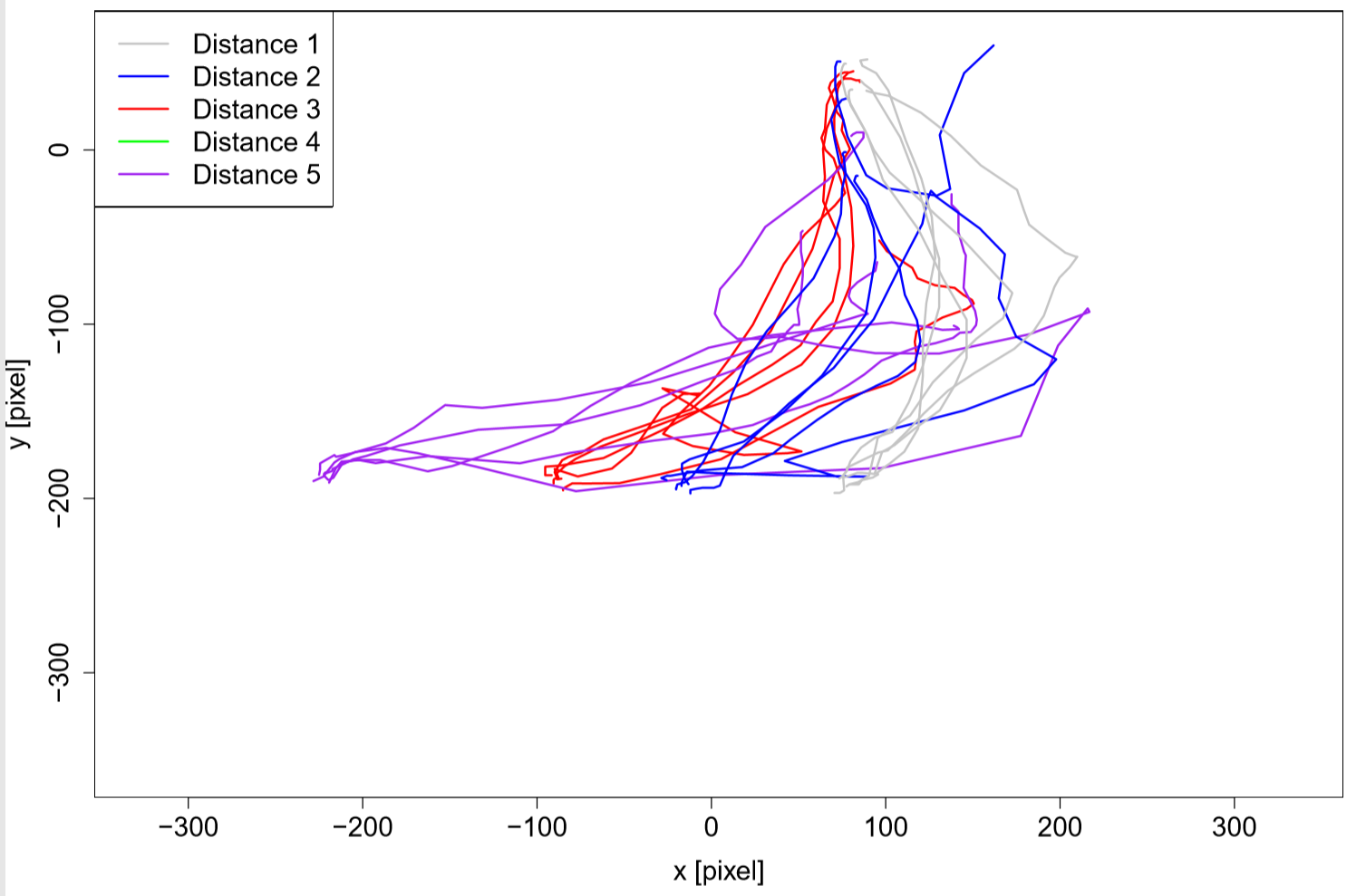

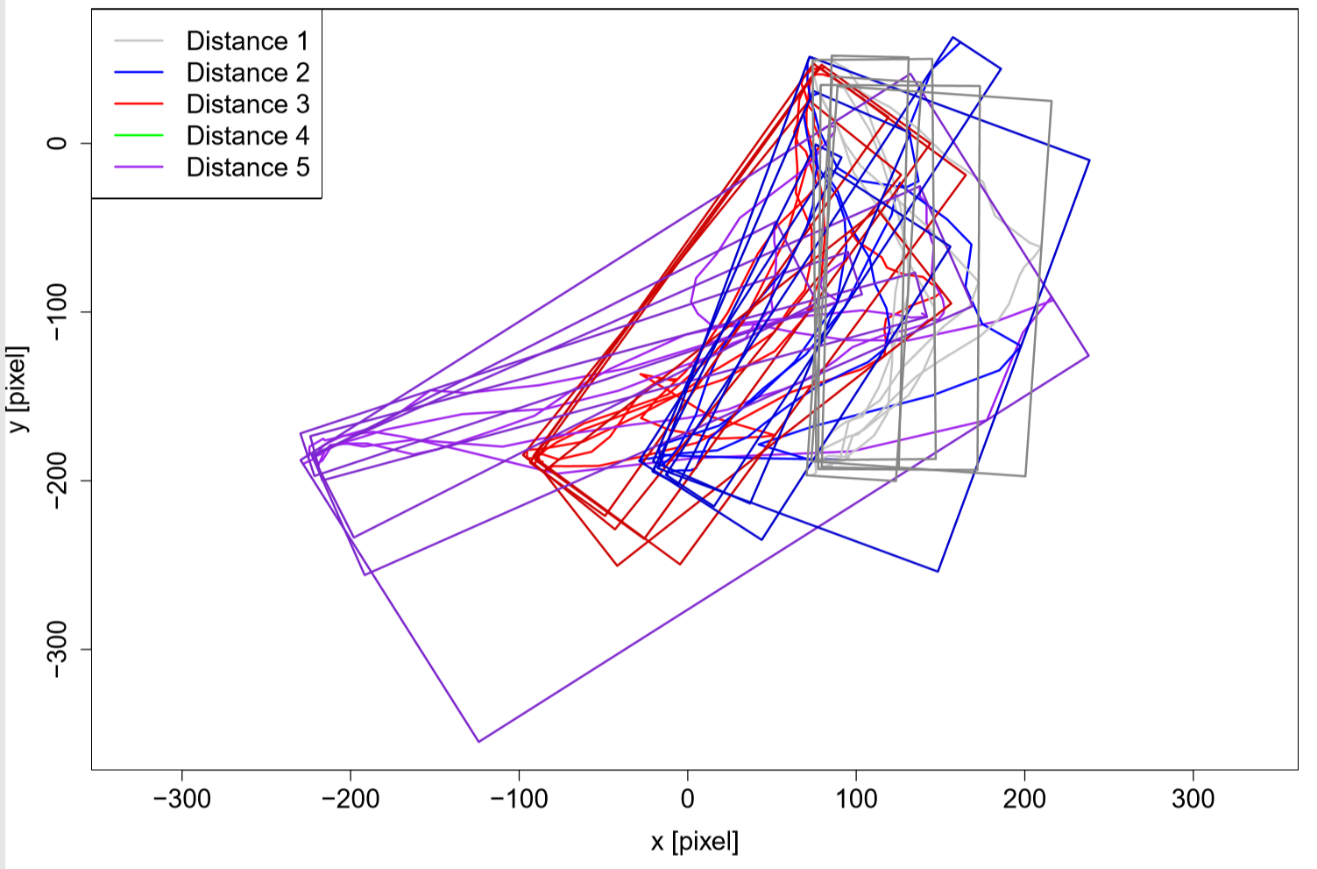


Figure 5: Top: trajectories for all five distances extracted from the videos for participant 6. The start position of the participants hand is on the upper right corner, the stop positions of the gripper at the bottom row. Bottom: additional oriented bounding boxes for every trajectory.


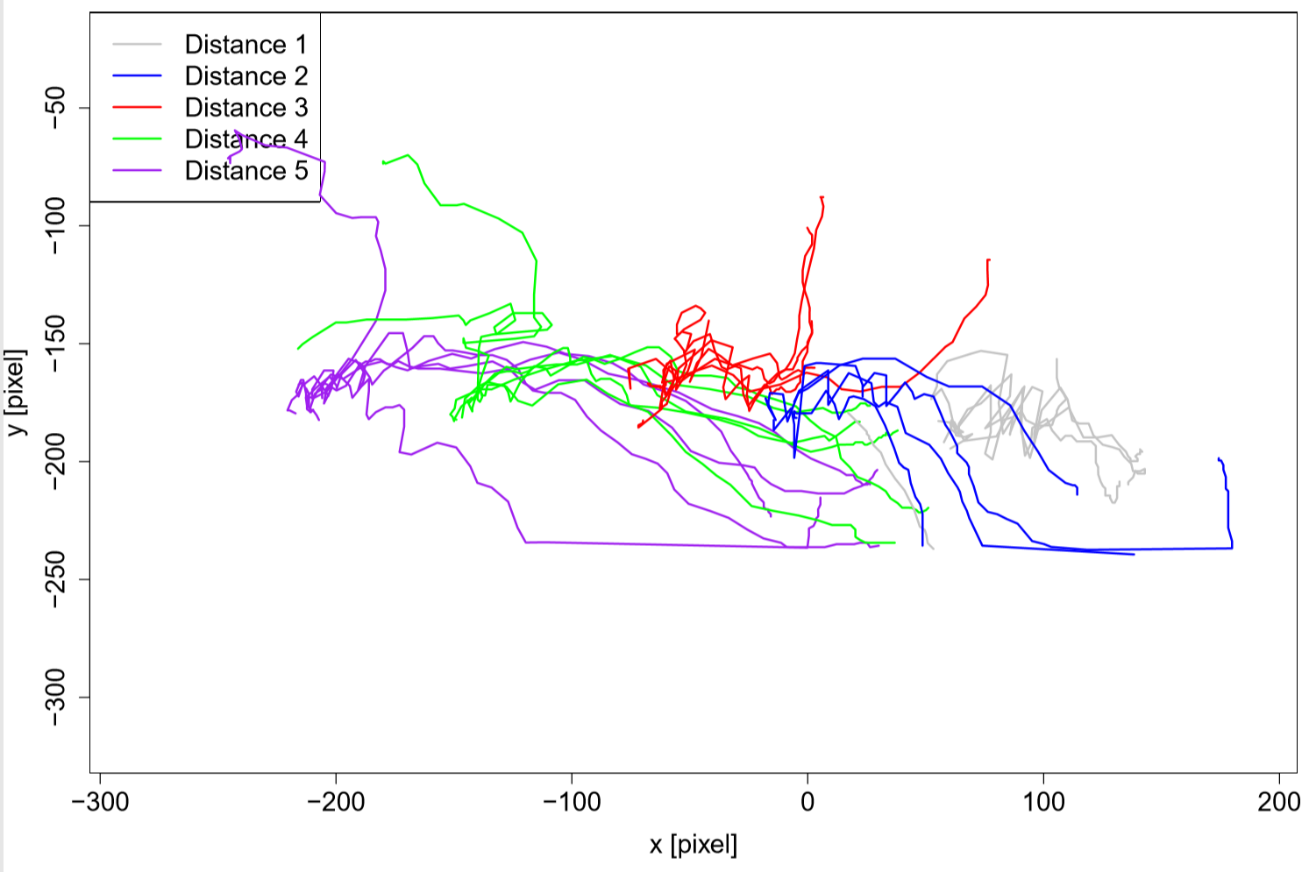

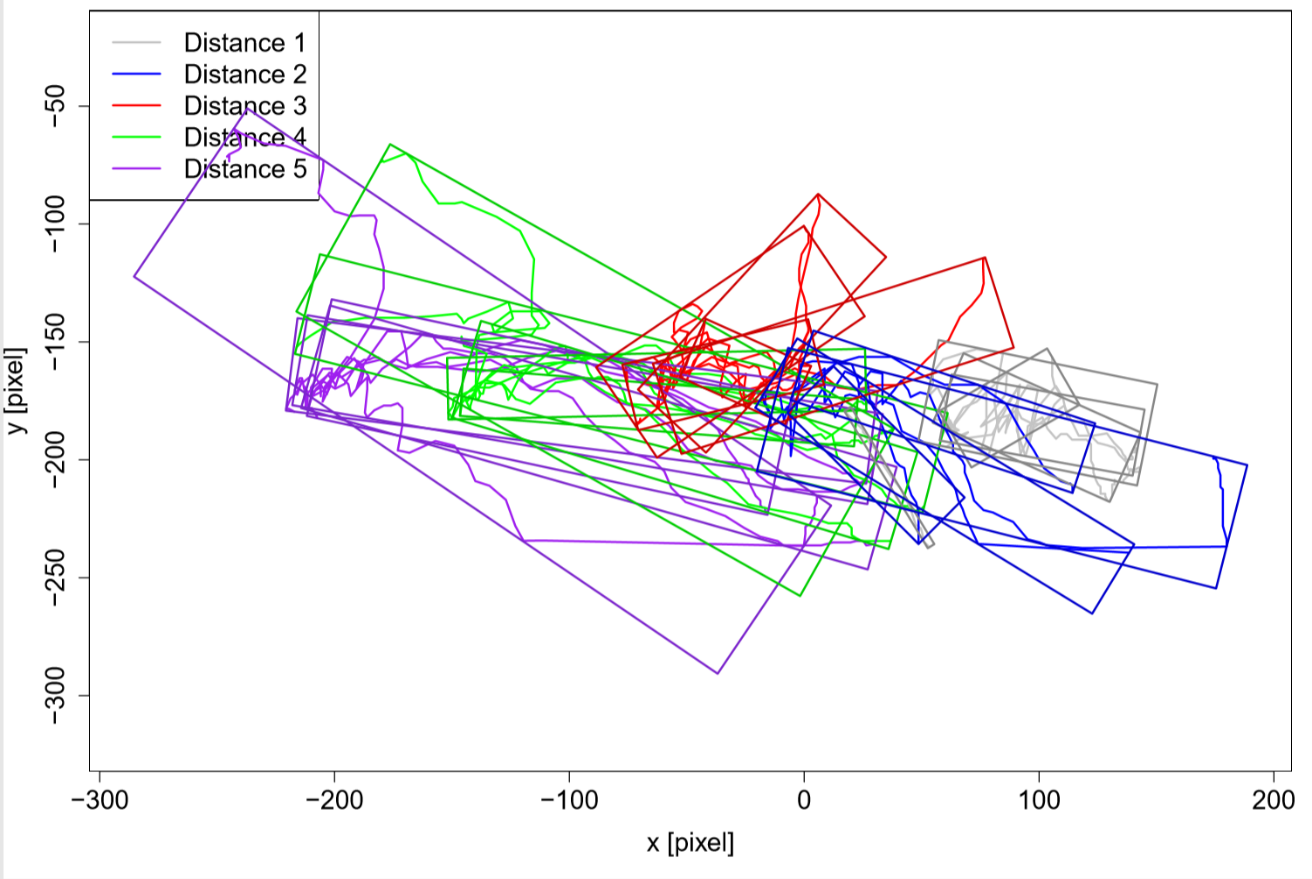


Figure 6: Top: trajectories for all five distances extracted from the videos for participant 7. The start position of the participants hand is on the upper right corner, the stop positions of the gripper at the bottom row. Bottom: additional oriented bounding boxes for every trajectory.


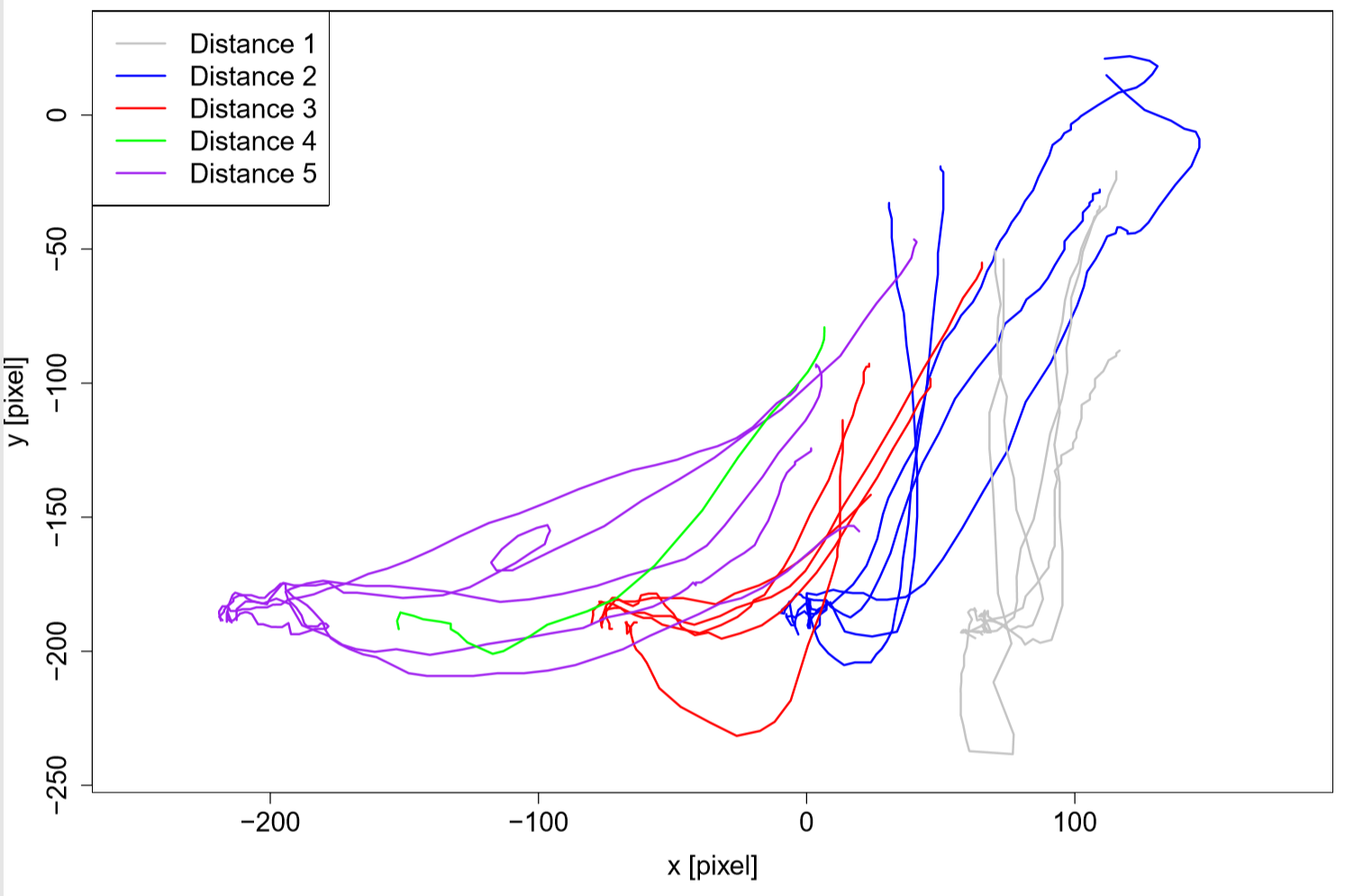

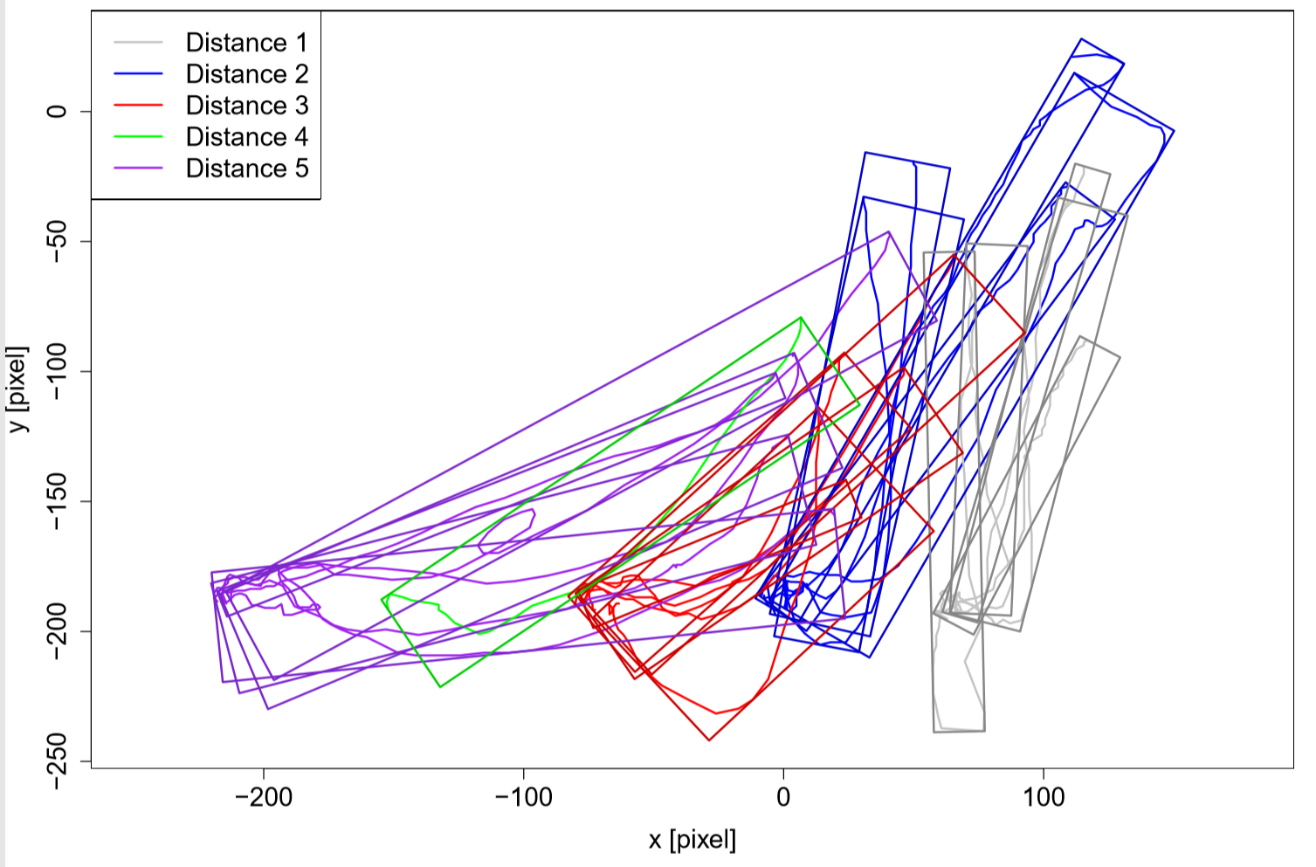


Figure 7: Top: trajectories for all five distances extracted from the videos for participant 9. The start position of the participants hand is on the upper right corner, the stop positions of the gripper at the bottom row. Bottom: additional oriented bounding boxes for every trajectory.


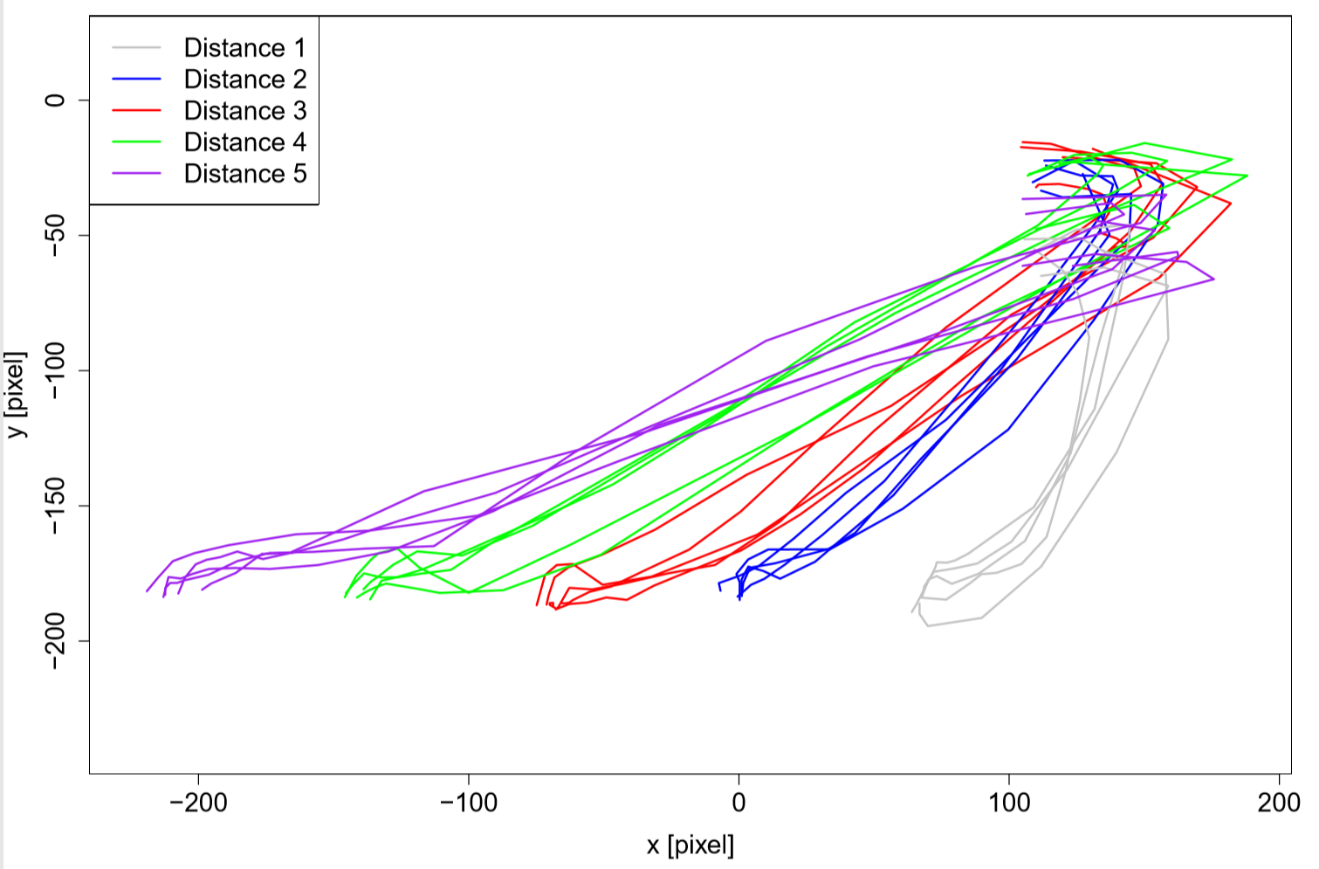

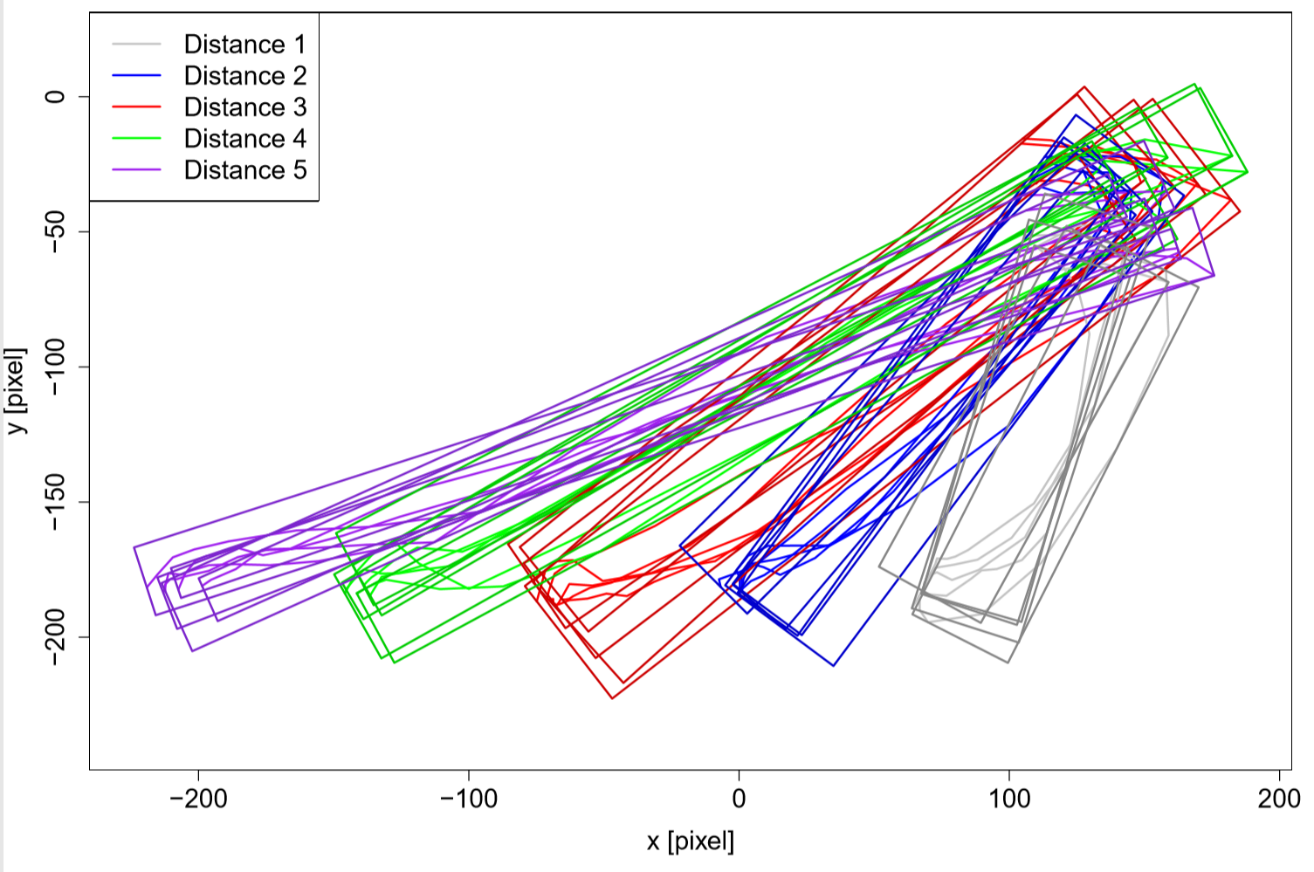


Figure 8: Top: trajectories for all five distances extracted from the videos for participant 10. The start position of the participants hand is on the upper right corner, the stop positions of the gripper at the bottom row. Bottom: additional oriented bounding boxes for every trajectory.

Table 1: Average width of oriented bounding boxes for all participants and each distance.

| Participant | Distance 1 [pixel] | Distance 2 [pixel] | Distance 3 [pixel] | Distance 4 [pixel] | Distance 5 [pixel] |
| --- | --- | --- | --- | --- | --- |
| 1 | 33,95 ± 25.64 | 47,25 ± 18.02 | 56,54 ± 29.99 | 58,00 ± 32.25 | 52,37 ± 13.73 |
| 2 | 25,09 ± 8.39 | 37,99 ± 43.29 | 40,01 ± 18.88 | 58,51 ± 51.01 | 29,15 ± 8.48 |
| 3 | - | 40,32 ± 15.74 | - | 37,87 ± 37.87 | 62,52 ± 22.15 |
| 4 | 28,40 ± 10.09 | 28,07 ± 11.09 | 34,78 ± 17.01 | 27,76 ± 4.55 | 32,57 ± 9.61 |
| 5 | 30,77 ± 13.43 | 24,47 ± 5.91 | 25,84 ± 6.22 | 34,92 ± 18.66 | 29,73 ± 15.05 |
| 6 | 78,31 ± 33.25 | 75,51 ± 62.70 | 76,69 ± 19.78 | - | 76,62 ± 70.95 |
| 7 | 27,06 ± 14.78 | 36,81 ± 11.76 | 36,30 ± 8.21 | 42,83 ± 23.89 | 52,02 ± 19.67 |
| 8 | - | - | - | - | - |
| 9 | 20,51 ± 5.28 | 31,99 ± 10.58 | 40,07 ± 17.43 | 40,57 ± NA | 36,70 ± 15.03 |
| 10 | 39,96 ± 2.36 | 33,99 ± 9.73 | 44,99 ± 6.38 | 26,43 ± 7.86 | 20,67 ± 6.57 |

Table 2: Average time difference between stop of gripper and start of grasping movement for all participants and each distance. Negative values state that the human grasp started before the robot movement stopped.

| Participant | Distance 1 [s] | Distance 2 [s] | Distance 3 [s] | Distance 4 [s] | Distance 5 [s] |
| --- | --- | --- | --- | --- | --- |
| 1 | -1,27 ± 0.79 | -1,40 ± 2.43 | -1,84 ± 1.06 | -1,43 ± 1.02 | -0,40 ± 2.10 |
| 2 | - | - | - | - | - |
| 3 | - | -0,42 ± 0.50 | - | -0,4 ± 0.86 | -0,39 ± 0.86 |
| 4 | 0,08 ± 0.31 | 0,27 ± 0.33 | -0,79 ± 1.01 | -0,02 ± 0.14 | 0,17 ± 0.38 |
| 5 | -2,19 ± 1.41 | -1,24 ± 0.57 | -0,27 ± 1.14 | -0,36 ± 1.31 | -0,77 ± 0.89 |
| 6 | -0,80 ± 0.49 | -0,81 ± 0.36 | -0,96 ± 0.62 | - | -1,09 ± 0.42 |
| 7 | -1,75 ± 2.27 | -2,60 ± 2.21 | 0,12 ± 1.28 | -0,94 ± 1.19 | -2,86 ± 1.94 |
| 8 | - | - | - | - | - |
| 9 | -2,41 ± 0.55 | -2,57 ± 0.69 | -1,69 ± 0.71 | -1,67 ± NA | -1,95 ± 1.23 |
| 10 | -0,55 ± 0.32 | -0,27 ± 0.23 | -0,29 ± 0.61 | -0,15 ± 0.72 | -0,10 ± 0.40 |
